# Transplantation of the LRP1^high^ subpopulation of human umbilical cord-derived mesenchymal stem cells improves ovarian function in mice with premature ovarian failure and aged mice

**DOI:** 10.1186/s13287-024-03660-0

**Published:** 2024-03-05

**Authors:** Jiacheng Shen, Li Wu, Xiaoying Shi, Gang Chen, Tingwei Liu, Fangfang Xu, Xiaocui Xu, Xiaochen Kou, Yanhong Zhao, Hong Wang, Chenfei Wang, Shaorong Gao, Shaohua Xu

**Affiliations:** 1 Shanghai Key Laboratory of Maternal Fetal Medicine, Clinical and Translational Research Center of Shanghai First Maternity and Infant Hospital, School of Life Sciences and Technology, Tongji University, Shanghai, 200092 China; 2 Key Laboratory of Spine and Spinal Cord Injury Repair and Regeneration of Ministry of Education, Department of Orthopedics, Tongji Hospital, School of Life Science and Technology, Tongji University, Tongji, 200092 China; 3 Frontier Science Center for Stem Cell Research, School of Life Sciences and Technology, Tongji University, Shanghai, 200092 China

**Keywords:** Premature ovarian failure, Ovarian function, Human umbilical cord-derived mesenchymal stem cells, Transplantation, LRP1, Aging

## Abstract

**Background:**

Premature ovarian failure (POF) has a profound impact on female reproductive and psychological health. In recent years, the transplantation of umbilical cord-derived mesenchymal stem cells (UC-MSCs) has demonstrated unprecedented potential in the treatment of POF. However, the heterogeneity of human UC-MSCs remains a challenge for their large-scale clinical application. Therefore, it is imperative to identify specific subpopulations within UC-MSCs that possess the capability to improve ovarian function, with the aim of reducing the uncertainty arising from the heterogeneity while achieving more effective treatment of POF.

**Methods:**

10 × Genomics was performed to investigate the heterogeneity of human UC-MSCs. We used LRP1 as a marker and distinguished the potential therapeutic subpopulation by flow cytometry, and determined its secretory functions. Unsorted UC-MSCs, LRP1^high^ and LRP1^low^ subpopulation was transplanted under the ovarian capsules of aged mice and CTX-induced POF mice, and therapeutic effects was evaluated by assessing hormone levels, estrous cycles, follicle counts, and embryo numbers. RNA sequencing on mouse oocytes and granulosa cells after transplantation was performed to explore the mechanism of LRP1^high^ subpopulation on mouse oocytes and granulosa cells.

**Results:**

We identified three distinct functional subtypes, including mesenchymal stem cells, multilymphoid progenitor cells and trophoblasts. Additionally, we identified the LRP1^high^ subpopulation, which improved ovarian function in aged and POF mice. We elucidated the unique secretory functions of the LRP1^high^ subpopulation, capable of secreting various chemokines, cytokines, and growth factors. Furthermore, LRP1 plays a crucial role in regulating the ovarian microenvironment, including tissue repair and extracellular matrix remodeling. Consistent with its functions, the transcriptomes of oocytes and granulosa cells after transplantation revealed that the LRP1^high^ subpopulation improves ovarian function by modulating the extracellular matrix of oocytes, NAD metabolism, and mitochondrial function in granulosa cells.

**Conclusion:**

Through exploration of the heterogeneity of UC-MSCs, we identified the LRP1^high^ subpopulation capable of improving ovarian function in aged and POF mice by secreting various factors and remodeling the extracellular matrix. This study provides new insights into the targeted exploration of human UC-MSCs in the precise treatment of POF.

**Graphical abstract:**

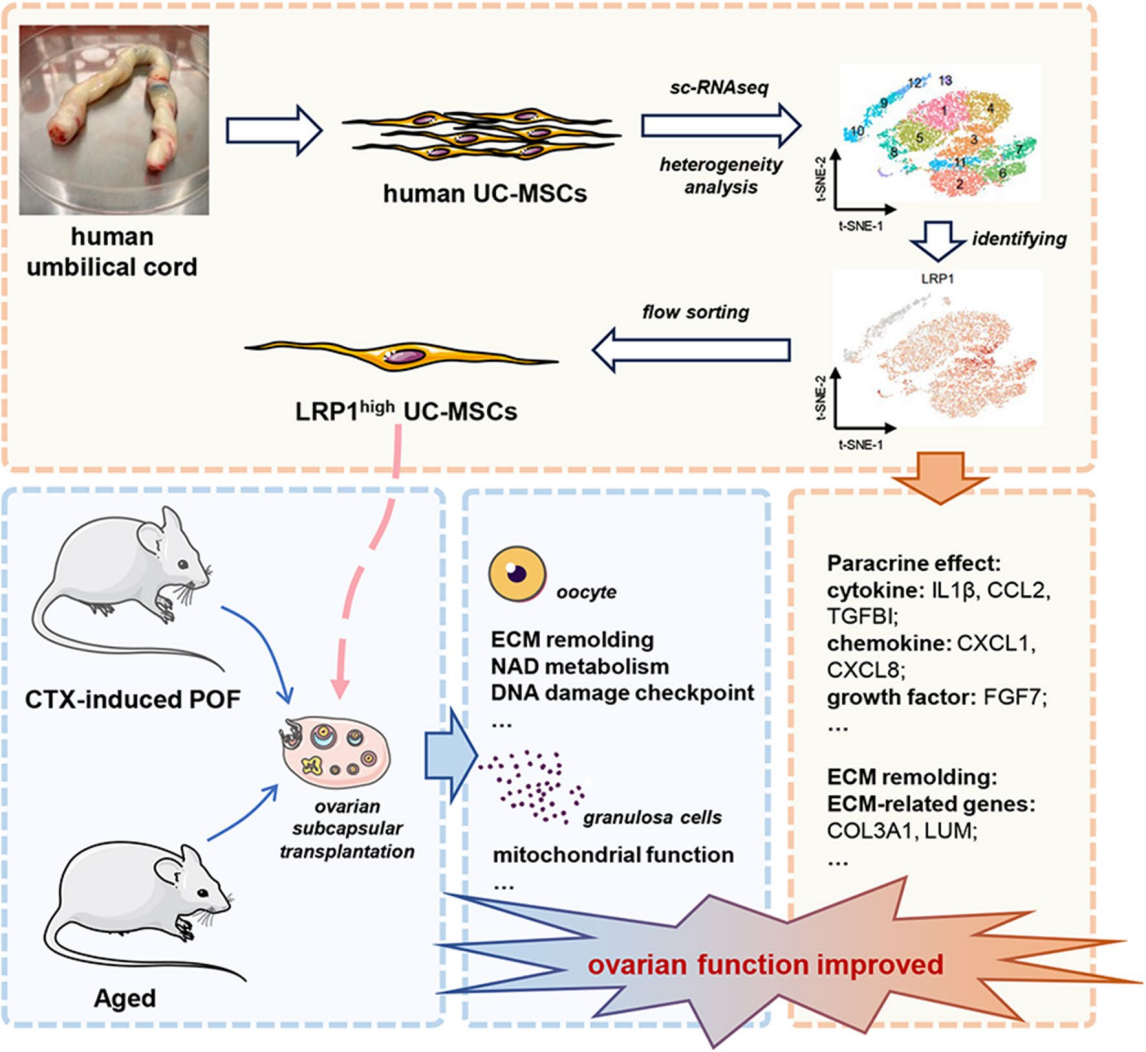

**Supplementary Information:**

The online version contains supplementary material available at 10.1186/s13287-024-03660-0.

## Introduction

Premature ovarian failure (POF) is a debilitating condition that substantially affects female reproductive function and psychological well-being [1, 2]. According to epidemiological data, the global incidence of POF ranges from 1 to 3% and has been increasing in recent years, with a trend towards younger age groups [3]. Currently, hormone replacement therapy (HRT) is the primary clinical approach for treating POF. However, HRT only provides temporary relief of menopausal symptoms and cannot effectively reverse ovarian failure [4]. With the advancement of regenerative medicine, stem cells, as a population of undifferentiated cells, have emerged as a promising avenue for POF treatment [5–8]. Increasing clinical evidence suggests that mesenchymal stem cells, particularly umbilical cord-derived mesenchymal stem cells (UC-MSCs), are an ideal cell source for clinical POF therapy due to their low tumorigenicity, low immunogenicity, high proliferation rate, and easy accessibility [9–12].

Despite the major advantages of UC-MSCs in POF research, their heterogeneity limits the study and application of UC-MSCs [13, 14]. Some studies suggest that regardless of donor and passage, human UC-MSCs exhibit limited heterogeneity [15]. However, most studies indicate heterogeneity in the proliferative capacity, differentiation potential, and immunomodulatory characteristics of human UC-MSCs [16–18]. A study investigating the therapeutic effects of human UC-MSC heterogeneity in POF demonstrated that CD146^+^ UC-MSCs possess advantages in immune regulation and cell proliferative characteristics. However, in terms of improving ovarian function, there was no significant difference between the CD146^+^ and CD146^−^ subpopulations [19]. These studies suggest that the heterogeneity of UC-MSCs has an impact on clinical efficacy. Yet, specific subpopulations of UC-MSCs that are effective in improving ovarian function have not been thoroughly investigated at present. Therefore, screening and identifying a specific subpopulation that can efficiently improve ovarian function will have significant clinical implications for the treatment of POF with UC-MSCs.

In our study, we revealed the heterogeneity of human UC-MSCs using single-cell sequencing technology (10 × Genomics). Through in vitro and in vivo experiments, it was determined that the LRP1^high^ subpopulation has therapeutic effects in improving ovarian function. This finding provides theoretical support for the screening of stem cell preparations for use in POF, aiming to reduce the impact of other interfering cell components on ovarian tissue and provide precise, safe, and effective clinical strategies for stem cell therapy in POF. Mechanistic exploration further revealed that the LRP1^high^ subpopulation primarily improves the mitochondrial function of granulosa cells by modulating DNA damage pathways, extracellular matrix-related signals, and cellular metabolism. Consequently, this treatment more effectively enhances the quality of aged mouse oocytes, offering new insights into targeting the LRP1^high^ subpopulation for precise treatment of premature ovarian failure.

## Materials and methods

### Isolation and culture of human UC-MSCs

The two human umbilical cord samples from full-term newborns were collected from the Shanghai First Maternal and Infant Hospital, and informed consent was obtained (ethical certification number: KS1956) with permission from the Medical Ethics Committee of Shanghai First Maternity and Infant Hospital. Human UC-MSCs were isolated using the adherent method. The umbilical cord was rinsed with DPBS (Gibco, USA) and cut into approximately 5 mm^3^ tissue fragments after removing the veins and arteries. Subsequently, fragments were dispersed in a 10 cm dish coated with 0.1% gelatin (Millipore, USA). After 6 h, α-MEM (Gibco) complete culture medium containing 5% UltraGRO-advanced (AventaCell BioMedical, USA), penicillin/streptomycin (Gibco) and heparin (Anhui, China) was added, and the dishes were placed in a 37 °C, 5% CO_2_ incubator until human UC-MSCs migrated out from the tissue. During the passage 0 (P0) culture period, an appropriate amount of culture medium was added to the dishes every two days to maintain a good condition. Approximately 10 days later, UC-MSCs were digested using 0.05% trypsin–EDTA (Gibco), and they were passaged in a new cell culture dish at a ratio of 1:3. UC-MSCs were cryopreserved using a serum-containing freezing medium containing 50% α-MEM, 40% FBS (Gibco), and 10% DMSO (Sigma-Aldrich, USA) and stored in liquid nitrogen for long-term preservation. The human UC-MSCs used for single-cell sequencing in this study were from the sixth passage (P6), while human UC-MSCs from passages earlier than P9 were used for cellular and animal experiments.

### Culture of the KGN human ovarian granulosa cell tumor line

The human ovarian granulosa cell tumor cell line KGN was obtained from the American Type Culture Collection (ATCC, Manassas, USA). The cells were cultured in DMEM/F12 (Gibco) with 10% FBS and penicillin/streptomycin and maintained in a 37 °C, 5% CO_2_ incubator. KGN cells were digested using 0.05% trypsin–EDTA and cryopreserved using serum-free freezing medium.

For experiments, KGN cells were seeded at a density of 5 × 10^4^ cells per 100 μL in 6-well plates. After adherence, the cells were pretreated with complete medium containing 1 mg/mL cyclophosphamide (CTX, C0768, Sigma-Aldrich, USA) for 3 h. Subsequently, each subpopulation of human UC-MSCs was seeded at a density of 3 × 10^4^ cells per 100 μL in the upper chamber, followed by coculture for 24 h. Quantitative reverse transcription PCR (qRT‒PCR) was performed to assess the expression of target genes.

### Flow cytometry analysis and sorting

Human UC-MSCs were collected and evenly distributed and then resuspended in 100 μL of FACS buffer (DPBS + 5% BSA (Sigma-Aldrich, USA)). Antibody staining was performed at a concentration of 5 μg/10^6^ cells using the following antibodies: CD73-FITC (Biolegend, USA, 344015), CD90-APC (Biolegend, USA, 328113), CD13-APC (Biolegend, USA, 301705), CD34-FITC (Biolegend, USA, 343503), CD29-PE (Biolegend, USA, 303004), CD105-PE (Biolegend, USA, 800503), CD45-PE/Cy7 (Biolegend, USA, 304015), CD19-PE/Cy7 (Biolegend, USA, 302215), PLAU-APC (Biolegend, USA, 369004), KRT19-Alexa Fluor 647 (BD Biosciences, USA, 563648), and CD91-PE (BD Biosciences, USA, 550497). The staining was carried out on ice protected from light for 30 min. After staining, the cells were washed with FACS buffer to remove excess antibodies, followed by filtration. Flow cytometry analysis was performed within 30 min to identify the primary UC-MSCs. For the sorting of specific subpopulations of human UC-MSCs, 1–2 mL of complete culture medium was added to a centrifuge tube to receive human UC-MSCs expressing high levels (LRP1^high^) or low levels (LRP1^low^) of LRP1. The criteria for categorizing into LRP1^high^ and LRP1^low^ are as follows: the top 25% of LRP1-positive cells are designated as the LRP1^high^ subpopulation, while the bottom 25% of LRP1-positive cells are classified as the LRP1^low^ subpopulation. UC-MSCs between these two subpopulations were subsequently excluded. The collected cells were then plated in a culture dish and incubated overnight. The next day, the cells were subjected to experimental analysis or transplanted into the ovarian bursa of mice.

### Enzyme-linked immunosorbent assay (ELISA)

Following cell counting, human UC-MSCs were seeded in 6-well plates at the same cell density. After 24 h of incubation, the supernatant was collected and centrifuged at 1000×*g* for 10 min. The target proteins in the supernatant were detected using human CXCL8 and IL-1β ELISA kits (ABclonal, Shanghai, China), following the manufacturer's instructions. The remaining supernatant was stored at -80 °C to prevent repeated freeze‒thaw cycles.

### Total RNA extraction, reverse transcription, and quantitative PCR

For cell line cells, a chemical extraction method was used. The cells were collected and lysed by adding 1 mL of TRIzol (TaKaRa, Japan) followed by vigorous pipetting. Then, 200 μL of chloroform was added, mixed by vortexing, and allowed to separate into layers. The mixture was centrifuged at 4 °C and 12,000 rpm for 15 min, and the supernatant was transferred to a new 1.5 mL centrifuge tube. An equal volume of isopropanol (Shanghai, China) was added, mixed by inversion, and left undisturbed for 30 min. After centrifugation at 4 °C and 12,000 rpm for 10 min, the supernatant was discarded, and the pellet was washed twice with 1 mL of 75% ethanol (Shanghai, China), followed by repeated centrifugation. The pellet was then dissolved in 9 μL of RNase-free water (ABM, Canada), and the RNA concentration was determined. For mouse oocytes, a PicoPure RNA Isolation Kit (Thermo Fisher Scientific, USA) was used for extraction of trace amounts of oocyte RNA according to the manufacturer's instructions. Subsequently, 1 μg of total RNA was reverse transcribed using the 5 × All-in-one kit (ABM, Canada) according to the manufacturer's instructions. The resulting cDNA was appropriately diluted and subjected to quantitative PCR using Premix Ex Taq™ (TaKaRa, Japan), and ΔCT values were calculated for expression analysis. The sequences of the primers used in this study are shown in Additional file 7: Table S6.

### H&E staining of tissues

Mouse organ tissues were fixed with 4% PFA (Servicebio, Wuhan, China) and dehydrated using a graded ethanol series, followed by clearing with xylene (Shanghai, China). The pretreated tissues were embedded in paraffin (Sigma-Aldrich, USA), and once the paraffin blocks solidified, they were trimmed and sectioned into 5 μm thick slices. The paraffin sections were then deparaffinized using xylene and a graded ethanol series, stained with hematoxylin–eosin (Servicebio, Wuhan, China), and finally mounted with neutral resin (Servicebio, Wuhan, China). The morphology of the tissues was observed under a microscope, and images were captured using a slide scanner.

### Measurement of mitochondrial membrane potential

For the detection of mitochondrial membrane potential in KGN cells, the cells were seeded in 35 mm confocal dishes. Mitochondrial membrane potential was assessed using the JC-1 Mitochondrial Membrane Potential Assay Kit (Beyotime, Shanghai, China) following the instructions provided. The cells were then observed, and images were captured using a laser confocal microscope.

### Animal experiments

5-week-old female and male ICR mice and 12-month-old female ICR mice were obtained from Shanghai Bikai Laboratory Animal Co., Ltd. All experimental mice were kept in SPF-grade animal facilities. 12-month-old mice were housed in SPF-level mouse facilities and raised until 18 months of age for experimental use. The experiment on mice was approved by the Animal Ethics Committee of Tongji University.

*Establishment of POF mouse model*. The method for establishing the CTX-induced POF mouse model is shown in Additional file 1: Fig. S1A. Female ICR mice at 6–8 weeks of age were intraperitoneally injected with 50 mg/kg CTX for 14 consecutive days, while the control group received an equivalent dose of DPBS. Vaginal smears were collected daily for one week to monitor the estrous cycle and assess ovarian function. After a week, peripheral blood was collected from the mice to measure serum levels of FSH, E2, AMH, LH, and P4 using ELISA kits (Mlbio, Shanghai, China), which indicated ovarian hormone function and ovarian reserve depletion, confirming the successful establishment of the POF mouse model.

*Transplantation of human UC-MSCs under the ovarian capsule in mice*. After the mice were anesthetized with an intraperitoneal injection of 2,2,2-tribromoethanol (T48402, Sigma-Aldrich, USA), the mice were placed ventral side down on a sterile operating table. Following disinfection of the surgical site, a 2–3 cm skin incision and a 0.5–1 cm incision of the muscle layer were made. Under a stereomicroscope, the ovaries were gently pulled out from the abdominal cavity. Subsequently, the ovarian capsule was lightly punctured with a 26 G needle, and 5 μL of human UC-MSCs suspended in DPBS at a concentration of 10^6^ cells was slowly injected beneath the ovarian capsule. The ovarian capsule was sealed using an electrocoagulation pen, and the ovaries were gently placed back into the abdominal cavity. The incisions in the muscle layer and skin were sutured with a thread.

*Intraorbital venous blood collection in mice*. The mice were immobilized to expose the eye for blood sampling. A 3 cm length 3 mm capillary tube was gently inserted into the intraorbital venous plexus. Blood was collected in a 1.5 mL centrifuge tube and left to stand for 15 min at 4 °C. The tube was then centrifuged at 3000 rpm for 20 min to separate the serum. The upper layer of serum was transferred to a new centrifuge tube and stored at − 80 °C for subsequent hormone analysis.

*Vaginal smears and estrous cycle determination in mice*. During the three weeks following stem cell transplantation, vaginal smears were collected from the mice every morning at 8 o'clock. The vaginal cavity was flushed with 20 μL of DPBS and gently blown 2–3 times, and the lavage fluid was spread onto a glass slide. Cell morphology was observed under a microscope to determine the stage of the estrous cycle. The proestrus stage is characterized by numerous small leukocytes, the estrus stage by irregularly shaped nucleated epithelial cells, the metestrus stage by cornified epithelial cells, and the diestrus stage by a few cornified epithelial cells and leukocytes. The complete number of cycles within the three-week period was recorded, and the number of days required for each estrous cycle was calculated.

*Ovarian follicle counting in mice*. After H&E staining, follicle counting was performed on mouse ovarian sections. The morphological criteria for follicles at different stages were as follows: (1) primordial follicle: surrounded by a layer of flattened granulosa cells or a mixture of flattened and cuboidal granulosa cells, with a total cell count of less than 7; (2) primary follicle: ≥ 7 cuboidal granulosa cells surrounding the oocyte; (3) secondary follicle: ≥ 2 layers of granulosa cells surrounding the oocyte; (4) early antral follicle: ≥ 2 layers but < 4 layers of granulosa cells surrounding the oocyte, with a follicular cavity diameter < 20 μm; and (5) antral follicle: a follicle with a clearly visible follicular cavity.

*Cohousing of mice and collection of E12.5 embryos*. Female and male mice were housed at a 1:2 ratio. The next morning, vaginal plugs were observed in female mice. The presence of a milky white solid plug at the vaginal orifice indicated successful mating. The mated female mice were placed in separate cages, and the day of plug observation was recorded as Day 1. On Day 12, the female mice were euthanized by cervical dislocation. Open the mouse abdomen to expose the uterus. From both sides of the uterine horns, incise the uterine muscle layer, sequentially retrieve the embryos, and place them in DPBS. The placentas attached to the embryos were removed, and the amniotic membranes were torn open to separate the E12.5 embryos. The well-developed embryos were counted and photographed.

*Collection of mouse oocytes and granulosa cells*. The ovaries of mice were dissected and placed in a 100 μL droplet of DPBS in a 10 cm dish on a heated stage. The surface of the ovaries was gently scraped with a 26 G needle to rupture the follicles and release oocytes and granulosa cells. Granulosa cells were isolated by digesting the cumulus-oocyte complexes with hyaluronidase (H1115000, Sigma-Aldrich, USA). The granulosa cells were then washed three times with 0.5% PBS-BSA, while the oocytes were transferred to a droplet of pronase solution (PRON-RO, Roche, Switzerland). After complete zona pellucida digestion, the oocytes were washed three times with 0.5% PBS-BSA. Finally, the granulosa cells and oocytes were separately collected in low-adsorption 200 μL centrifuge tubes for subsequent library construction.

### Single-cell sequencing and data analysis

*Library construction and sequencing for single-cell RNA-Seq*. Human UC-MSCs cultured up to the 6th passage were digested into a single-cell suspension, and the generation of single-cell gel beads (GEMs) was performed rapidly using the automated Chromium Controller system. The GEMs underwent reverse transcription, amplification, adapter ligation, and other steps, followed by 10 × Genomics single-cell transcriptome sequencing on the NovaSeq 6000 platform (Illumina) at Berry Genomics Co., Ltd., to analyze the heterogeneity of human UC-MSCs.

*Data processing and analysis for single-cell sequencing*. CellRanger (https://www.10xgenomics.com/) was used to align and annotate the raw sequencing data (fastq files) to obtain expression data. The cloupe file exported from the output data folder can be directly viewed using the professional software Loupe Cell Browser provided by 10X Genomics (https://www.10xgenomics.com/). Furthermore, the filtered_feature_bc_matrix.h5 file from the output data folder was imported into R software (version 4.0.5), and the Seurat (https://satijalab.org/) package's FindNeighbors and FindClusters functions were used to perform subpopulation clustering on the single-cell data. The RunUMAP function was used to visualize the dimensionally reduced results using UMAP. The FindAllMarkers function in the Seurat package was used to identify marker genes for each cell cluster, which serve as potential marker genes for cell type identification. Based on differentially expressed genes (DEGs) in each cluster and known cell markers reported in the literature, the cell types of human UC-MSCs were distinguished and annotated. The limma package was used for conventional screening of DEGs between different groups, with the following criteria: |FC|> 1.5 and adj.P.Value < 0.05. The Clusterprofile package was used for functional enrichment analysis of DEGs to elucidate the main functional differences between different groups. The analysis primarily focused on Gene Ontology (GO) terms, including biological process (BP), cellular component (CC), and molecular function (MF), as well as Kyoto Encyclopedia of Genes and Genomes (KEGG) pathways. Additionally, enrichment analysis of biological molecular pathways in the Reactome database and Wikipathways database was conducted to further explore relevant functions. The criteria for selecting biologically significant pathways were adj.P.Value < 0.05.

### RNA sequencing and data analysis

*Library construction and sequencing for RNA-Seq*. RNA sequencing of mouse oocytes and granulosa cells was performed using the Smart-seq2 method for library construction. The cells to be sequenced were lysed using cell lysis buffer, and the lysate was reverse transcribed into single-stranded DNA. The reverse transcription product was pre-amplified, purified using Ampure XP beads (Beckman, USA), and fragmented using a Covaris S220 instrument. The fragmented DNA was then recovered using the QIAquick PCR Purification Kit (QIAGEN, Germany), followed by end repair, A-tailing, and adapter ligation. Subsequent to two rounds of purification, the purified DNA was amplified after measuring its concentration. The amplified product was loaded onto a DNA gel for electrophoresis, and a gel block with band sizes ranging from 200 to 500 bp was selected. Gel extraction of the recovered DNA library was performed using the PCR Clean-up Gel Extraction Kit (Macherey–Nagel, Germany). The recovered DNA library was then subjected to high-throughput sequencing on the HiSeq 2500 platform (Illumina) at Berry Genomics Co., Ltd.

*Processing and analysis of RNA-Seq data*. The raw fastq data from the transcriptome sequencing were subjected to quality control and then aligned and annotated using HISAT2 and StringTie to generate an expression matrix. The transcriptomic expression data were imported into R software, and principal component analysis (PCA) was performed on the samples using the prcomp function. The distribution differences among the sample groups were visualized using the ggplot2 package. Differential expression analysis of genes between different groups was conducted using the limma package, and DEGs were selected based on the following criteria: |FC|> 1.5 and adj.P.Value < 0.05. Functional enrichment analysis of DEGs was performed using the clusterProfiler package, and functional terms with adj.P.Values < 0.05 were selected. The results were visualized using the ggplot2 package. DEGs obtained from the transcriptome sequencing of young and aged mouse oocytes were used to construct a scoring system for oocyte aging. This scoring system was then applied to evaluate the degree of oocyte aging in mice transplanted with different subtypes of UC-MSCs.

### Statistical analysis

The statistical data were analyzed and visualized using Microsoft Excel and GraphPad Prism 9 (GraphPad Software, Inc.). The analysis was performed by unpaired Student's t tests between two groups. All experiments were repeated at least three times.

## Results

### Heterogeneity analysis and subtype identification of human UC-MSCs

**Fig. 1:**
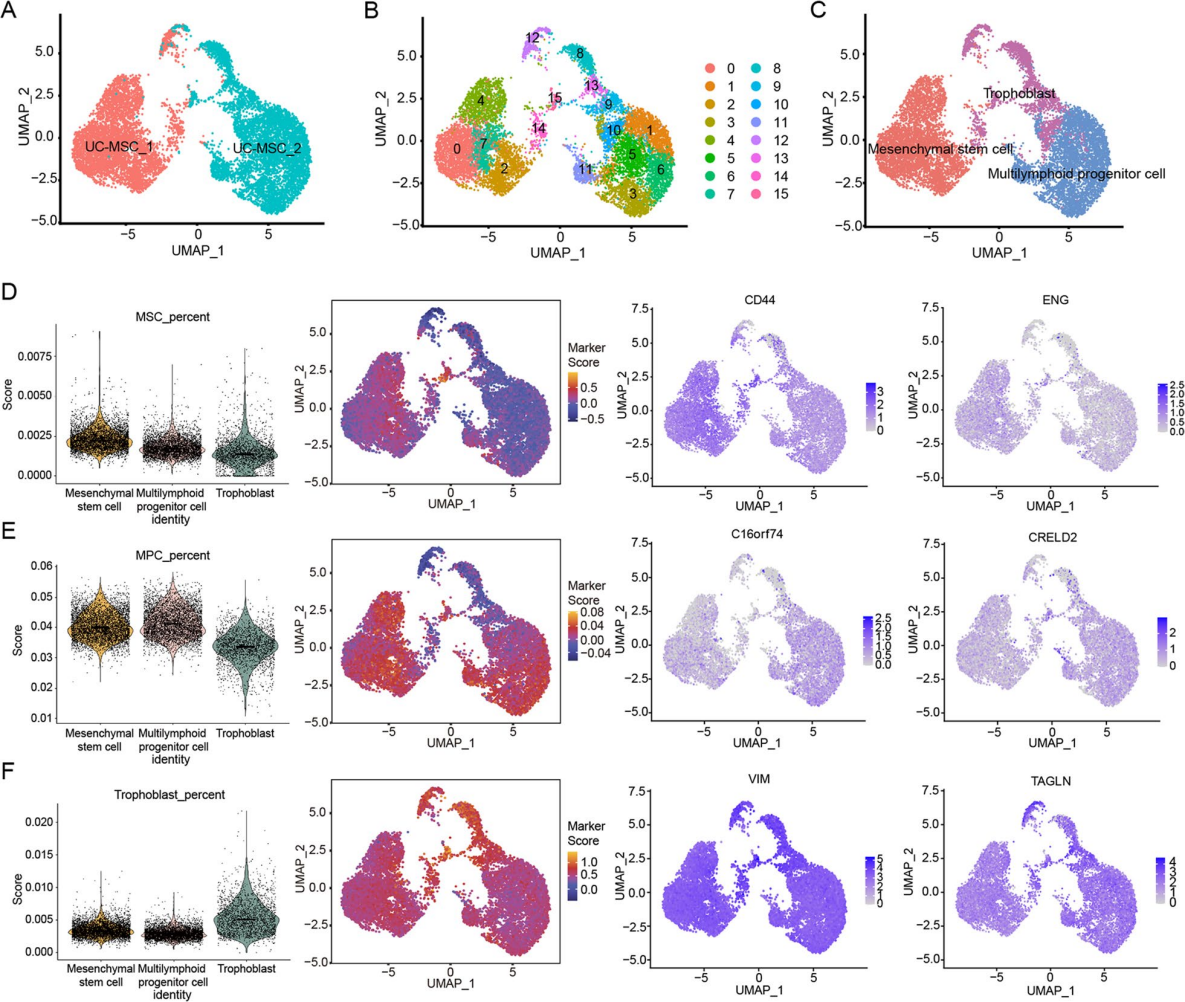
The heterogeneity of human UC-MSCs at single-cell resolution and subpopulations identification. **A** UMAP displays the clustering information of two cases of human UC-MSCs, distinguished by human UC-MSCs source (red for female human UC-MSCs, blue for male human UC-MSCs). **B** UMAP visualization of cluster information of 16 subclusters of two cases of human UC-MSCs. **C** UMAP visualization of three subtypes of two cases of human UC-MSCs. **D**–**F** Based on the expression of marker genes in each cell type, human UC-MSCs was divided into three cell subtypes: mesenchymal stem cells (MSCs, **D**), multilymphoid progenitor cell cells (MPCs, **E**), and trophoblast cells (**F**). The violin diagram shows the expression levels of the marker genes of each subtype in the three cell subtypes (left); the UMAP heatmap shows the expression abundance of marker genes of each subgroup in all cells (medium), with red indicating high level of expression and blue indicating low level of expression; UMAP feature plot shows the expression distribution of characteristic genes in each subtype (right), in which CD44 and ENG are the primary marker genes of MSCs, C16orf74 and CRELD2 are the marker genes representing MPCs, VIM and TAGLN represent trophoblast, and the depth of color indicates the level of gene expression

Human UC-MSCs were successfully isolated from umbilical cord tissues of newborn infants and highly expressed MSC markers, such as CD13, CD29, CD90, and CD105, and were negative for CD19, CD34, and CD45 (Additional file 1: Fig. S1B, C). To explore the heterogeneity of human UC-MSCs, two samples of human UC-MSCs were subjected to single-cell library construction and sequencing. We obtained a total of 11,735 high-quality single-cell transcriptomes from two samples covering 17,301 genes, with a median of 25,392 unique transcripts and 4,760 genes per cell from 2 samples (Fig. 1A). Sixteen clusters were identified based on the expression of differentially expressed genes (DEGs) in each cluster and known markers (Fig. 1B, Additional file 1: Fig. S2A and Additional file 2: Table S1), which were grouped into 3 main subtypes: mesenchymal stem cells (MSCs), multilymphoid progenitor cells (MPCs) and trophoblasts (Fig. 1C and Additional file 3: Table S2). MSCs were characterized by the expression of CD44, ENG, ITGB1, CD34, NT5E, MCAM, PECAM1, FZD9, THY1, and CD14 as marker genes and were enriched in functional terms such as cell aging, collagen metabolic process, positive regulation of cytokinesis and ECM-receptor interaction (Fig. 1D and Additional file 1: Fig. S2B-D). MPCs were identified by the expression of C16orf74 and CRELD2 (Additional file 4: Table S3) and enriched in terms such as embryonic development, cellular senescence and cellular response to TGF-β stimulus (Fig. 1E and Additional file 1: Fig. S2B, C, E). Trophoblast cells were marked by the expression of VIM, TAGLN, LVRN, and KRT7 (Fig. 1F and Additional file 1: Fig. S2B, C). These findings indicate the presence of substantial heterogeneity within UC-MSCs, with at least three distinct functional subtypes, and different subtypes may exhibit varying regenerative capacities, which could impact the effectiveness of ovarian functional restoration.

### LRP1^high^ is the subpopulation of human UC-MSCs with high secretory function

**Fig. 2:**
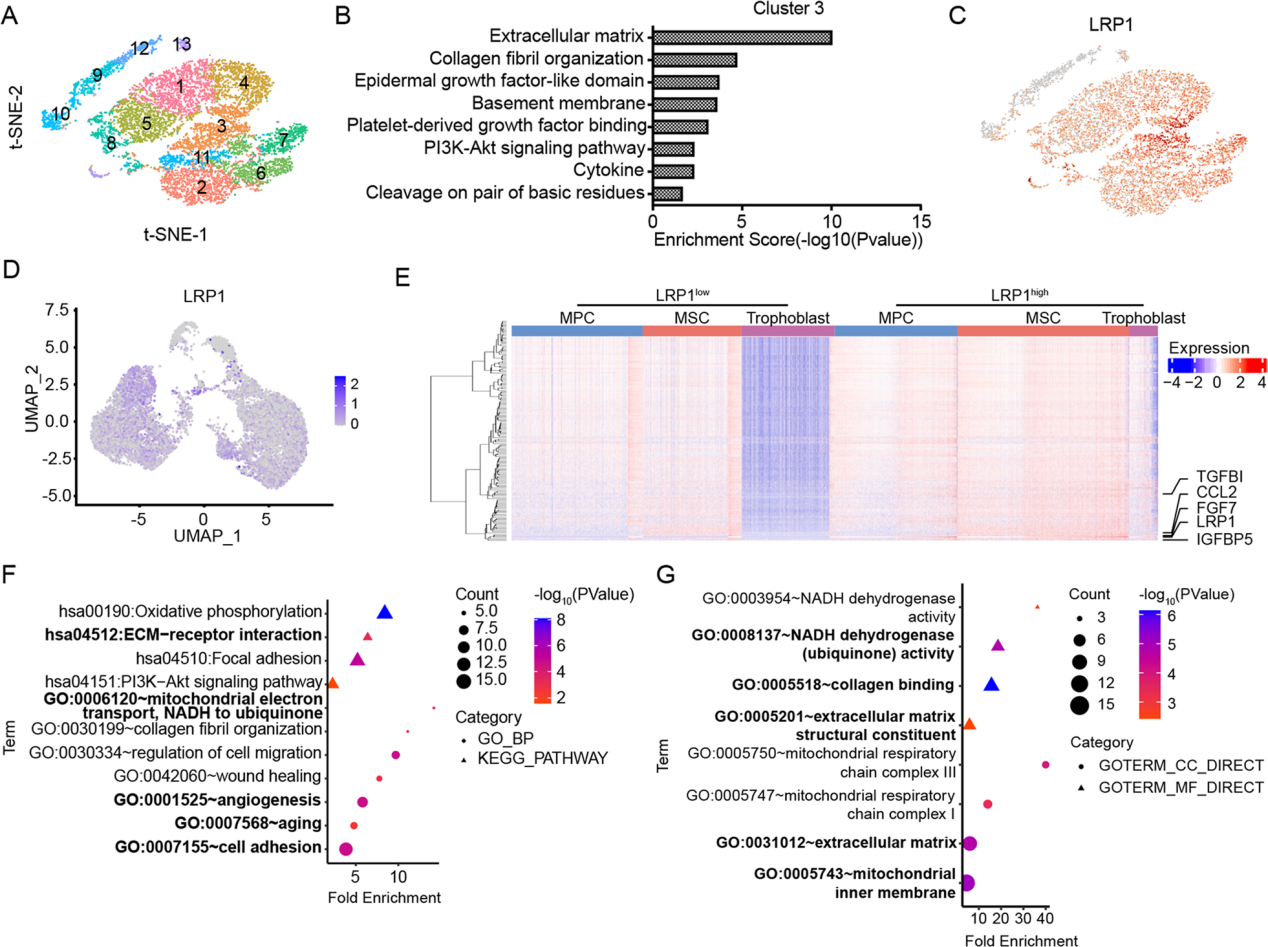
Identification and functional characterization of the LRP1^high^ subpopulation. **A–C** Based on cLoupe's Graph-Based clustering (**A**), it can be observed that cluster 3 possesses functions related to ECM regulation and secretion of various cytokines (**B**). According to the DEGs of this cluster of cells, cell surface marker LRP1 were selected for sorting (**C**). **D** UMAP feature plot shows the expression level and distribution of LRP1 in three cell subtypes. **E** Heatmap shows differentially expressed genes of LRP1^high^ and LRP1^low^ subpopulations. **F** Functional enrichment analysis (GO-BP and KEGG) of LRP1 related regulatory pathways. **G** Functional enrichment analysis (GO-CC and GO-MF) of LRP1 related regulatory pathways

To find the efficient cluster, we assessed the GO terms of the clusters based on “Graph-Based” and identified that Cluster III in t-SNE by cLoupe was enriched in extracellular matrix organization and chemotaxis (Fig. 2A, B). Here, based on the expression of DEGs, we selected surface protein LRP1 as the marker to perform flow cytometry sorting (Fig. 2C).

LRP1, as a member of the low-density lipoprotein receptor family, is involved not only in wound healing and tissue remodeling but also in regulating physiological processes such as early embryo implantation and development. Single-cell sequencing analysis revealed the LRP1 was predominantly expressed in the MSC subpopulation compared to the MPC and trophoblast subtypes (Fig. 2D). Based on the expression of LRP1, all cells were divided into two groups, LRP1^high^ and LRP1^low^, and further analysis was performed on the DEGs and their functions (Fig. 2E and Additional file 5: Table S4). GO-BP and KEGG pathway analysis indicated that LRP1 expression was mainly associated with aging, extracellular matrix interaction, cell adhesion, wound healing, collagen fibril formation, angiogenesis, mitochondrial electron transport (NADH to CoQ), and the PI3K-Akt signaling pathway (Fig. 2F). GO-CC and GO-MF functional analysis revealed that the LRP1^high^ subpopulation was primarily associated with NADH dehydrogenase activity, collagen binding, extracellular matrix and its structural composition, and mitochondrial respiratory chain complexes I/III (Fig. 2G).

**Fig. 3:**
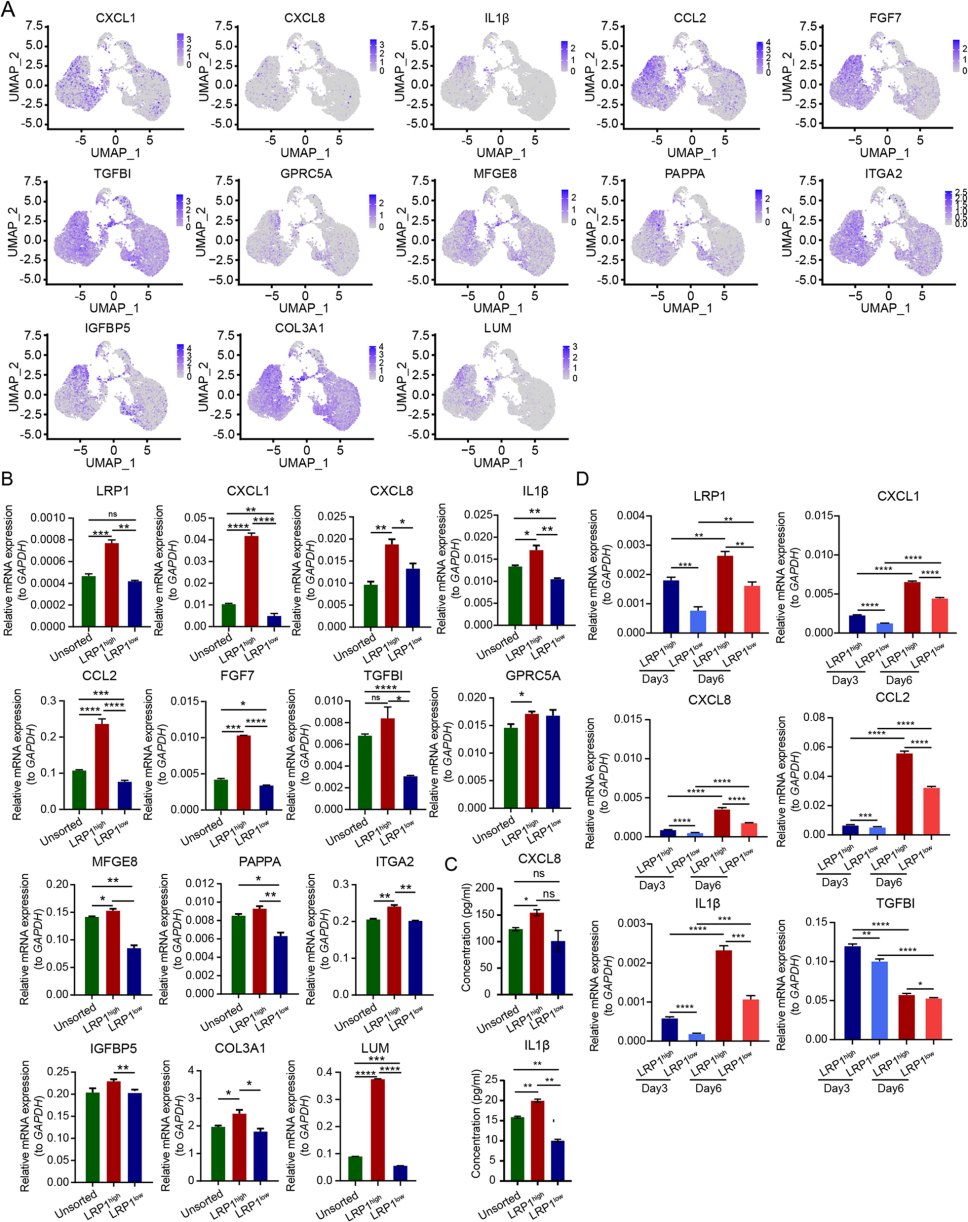
LRP1^high^ is the subpopulation with high secretory function. **A** UMAP feature plot shows the expression levels of genes related to the function regulated by LRP1 in human UC-MSCs. **B** The expression levels of LRP1, secreted factors and extracellular matrix related genes in different subpopulations were detected by qRT-PCR. Mean ± SD, ** P ≤ 0.01; * P ≤ 0.05. **C** The secretion levels of CXCL8 and IL1β in LRP1^high^ and LRP1^low^ subpopulations and unsorted human UC-MSCs were detected by ELISA. The expression levels of these two factors were highest in LRP1^high^ subpopulation and lowest in LRP1^low^ subpopulation. Mean ± SD, ** P ≤ 0.01; * P ≤ 0.05; ns: > 0.05. **D** qRT-PCR was used to detect the expression levels of LRP1 and secretory factors in each subpopulation of human UC-MSCs on the 3rd and 6th days after sorting. The sorted human UC-MSCs subgroups were able to maintain their functionality for a period of time. Mean ± SD, *n* = 3, **** P ≤ 0.0001; *** P ≤ 0.001; ** P ≤ 0.01; * P ≤ 0.05

Genes that were highly expressed in the LRP1^high^ subpopulation included various secretory factors, such as CXCL1, CXCL8, IL1β, CCL2, TGFBI, and FGF7, as well as numerous extracellular matrix-related genes, such as COL3A1 and LUM (Fig. 3A and Additional file 1: Fig. S3). Subsequently, the expression of secretory factors and extracellular matrix-related genes was validated by ELISA and qRT-PCR in UC-MSCs with different LRP1 expression levels. Consistent with the analysis of single-cell data, the mRNA expression of CXCL1, CXCL8, IL1β, CCL2, FGF7, TGFBI, GPRC5A, MFGE8, PAPPA, ITGA2, IGFBP5, COL3A1, and LUM in the LRP1^high^ subpopulation were higher than those in the LRP1^low^ subpopulation (Fig. 3B). The LRP1^high^ subpopulation showed a significant increase in the extracellular secretion of CXCL8 and IL1β compared to unsorted human UC-MSCs and the LRP1^low^ subpopulation (Fig. 3C). Moreover, on the 3rd and 6th days post-sorting, the LRP1^high^ subpopulation still maintained high levels of LRP1 and the extracellular secretory factors CXCL1, CXCL8, CCL2, IL1β, and TGFBI, suggesting that the sorted subpopulation can maintain functionality for a certain period (Fig. 3D). Consistent with our findings, the four human UC-MSCs samples from the GSE199071 dataset also demonstrated that the LRP1^high^ subpopulation possesses secretory functions for various factors including IL1β, FGF7, and plays a role in ECM regulation and mitochondrial function (Additional file 1: Fig. S4 and S5).

### The LRP1^high^ subpopulation significantly restored ovarian function in aged mice and CTX-induced POF mice

**Fig. 4:**
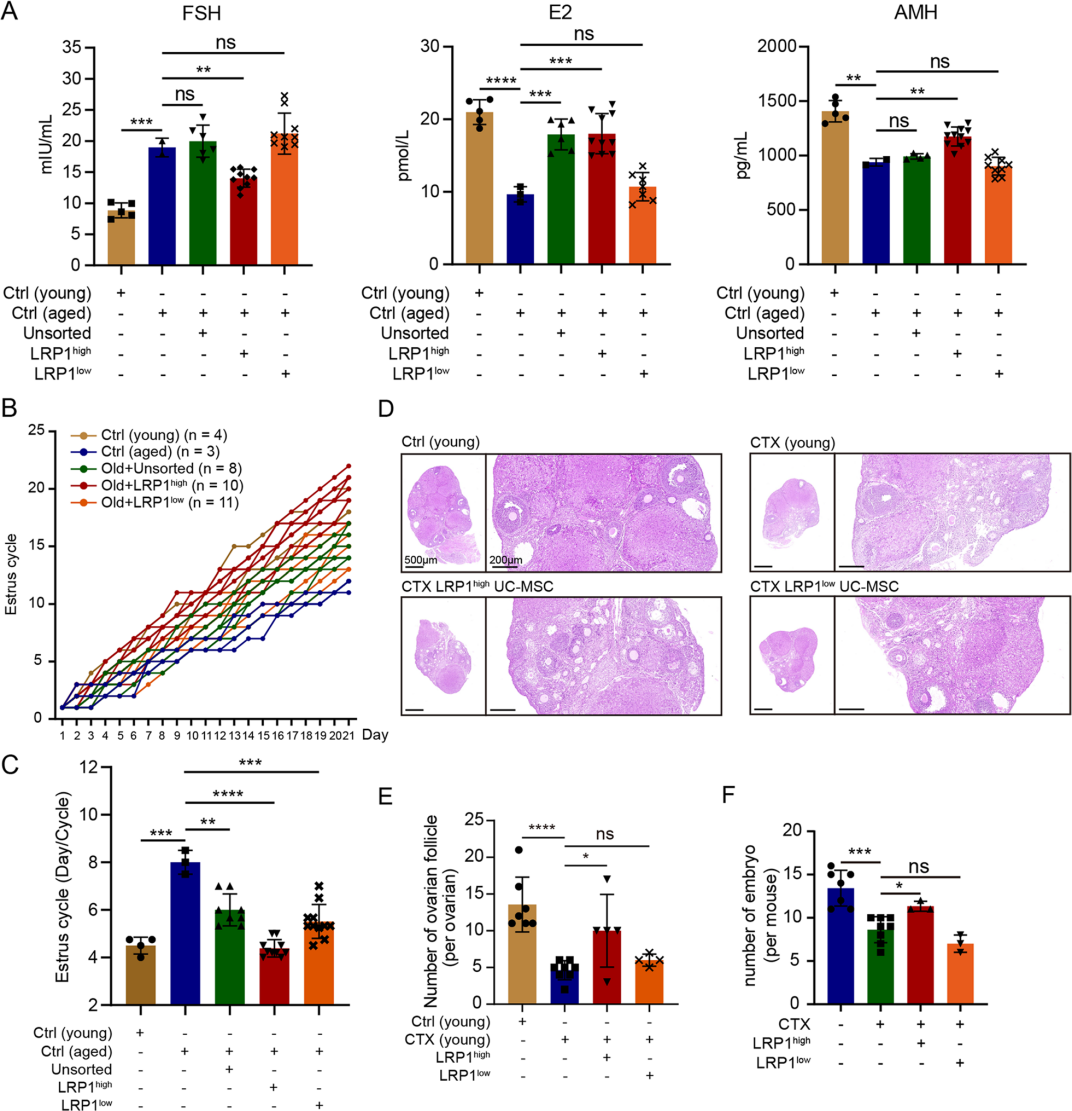
The LRP1^high^ subpopulation significantly restored ovarian function in aged mice and CTX-induced POF mice. **A** Changes of the serum hormone levels in aged mice after human UC-MSCs transplantation. Compared with LRP1^low^ subpopulation and unsorted human UC-MSCs, LRP1^high^ subpopulation can significantly restore serum LH, E2 and AMH levels in aged mice. The number of mice used was represented by different shaped dots in each group. Mean ± SD, **** P ≤ 0.0001; *** P ≤ 0.001; ** P ≤ 0.01; * P ≤ 0.05; ns: > 0.05. **B** Changes in estrous cycle of young mice, aged mice, and subpopulation treated aged mice. The entire estrous cycle was monitored for a duration of three weeks. The day of initial detection was labeled as "1," and each subsequent stage change was denoted by " + 1." If the stage remained the same as the previous day, the number from the previous day was retained, and this pattern continued for the full 21 days. **C** The total number of complete estrous cycles experienced by the mice within the three-week period was recorded, and the average duration of the estrous cycle was calculated for each group of mice. The number of mice used was represented by different shaped dots in each group. Mean ± SD, *** P ≤ 0.001; ** P ≤ 0.01; * P ≤ 0.05. **D** The H&E staining of the ovaries in CTX-induced POF mice after transplantation of subpopulations. **E** Statistical graph showing the changes in the number of ovarian follicles within the ovaries in **D**. The number of mice used was represented by different shaped dots in each group. **F** Quantification of E12.5 embryos in CTX-induced POF mice after transplantation of subpopulations. The number of mice used was represented by different shaped dots in each group

To determine the effects of the LRP1^high^ subpopulation on ovarian function, we transplanted LRP1^high^, LRP1^low^ and unsorted human UC-MSCs under the ovarian capsules. First, the safety of human UC-MSC transplantation was assessed by pathological sections of various organs in mice and the measurement of comprehensive blood indicators, and the results showed that human UC-MSC transplantation was safe and reliable in mice **(**Additional file 1: Fig. S6). Subsequently, LRP1^high^ and LRP1^low^ subpopulations, as well as unsorted human UC-MSCs, were transplanted under the ovarian capsule of 18-month-old naturally aged mice. Vaginal smears were collected daily for 3 weeks after transplantation. Compared to those of the 6- to 8-week-old young mice, the serum hormone levels of the 18-month-old aged mice exhibited a significant increase in serum FSH levels (P < 0.001), while E2 and AMH levels markedly decreased (E2: P < 0.0001; AMH: P < 0.01, Fig. 4A). This finding indicates a remarkable decline in ovarian function and a notable impairment of ovarian reserve in aged mice. After transplantation of unsorted human UC-MSCs, LRP1^high^, and LRP1^low^ subpopulations, the FSH levels in the mouse serum decreased to varying degrees, and E2 and AMH levels were restored. Specifically, in the aged mice transplanted with the LRP1^high^ subpopulation, the FSH level was dramatically decreased (P < 0.01), and there was a noteworthy increase in serum E2 levels (P < 0.001 Fig. 4A). Similarly, in the aged mice transplanted with unsorted human UC-MSCs, E2 levels were restored to an equivalent level (P < 0.001). In terms of ovarian reserve function, LRP1^high^ subpopulation significantly increased AMH levels in aged mice (< 0.001), indicating the restoration of ovarian reserve function (Fig. 4A). However, in the aged mice transplanted with unsorted human UC-MSCs and LRP1^low^ subpopulations, AMH levels were not restored. Furthermore, the estrous cycle was examined, and the results showed a significant prolongation of the estrous cycle in the 18-month-old aged mice compared to the 6–8-week-old young mice (P < 0.01). After UC-MSC transplantation, all three groups of mice showed significant recovery in the estrous cycle. Specifically, LRP1^high^ subpopulation significantly shortened the estrous cycle in aged mice (P < 0.0001, Fig. 4B, C**)**. In this study, fertility assessment was conducted in 18-month-old mice three weeks after transplantation of LRP1^high^ and LRP1^low^ subpopulations. The aged mice exhibited successful mating and vaginal plug formation, but no embryo development was observed in the uterus. In contrast, the aged mice transplanted with LRP1^high^ and LRP1^low^ subpopulations not only showed successful mating and vaginal plug formation but also exhibited several implanted embryos in the uterus. However, compared to those transplanted with the LRP1^low^ subpopulation, the aged mice transplanted with the LRP1^high^ subpopulation showed the presence of well-developed embryos in the uterine cavity **(**Additional file 1: Fig. S7A).

Previous studies have indicated that CTX, an alkylating agent, is widely used as an antitumor and immunosuppressive drug and is associated with the highest risk of POF [20, 21]. Chemotherapy-induced follicular and stromal cell senescence and apoptosis can lead to decreased ovarian weight, structural damage, atrophy, and alterations in hormone secretion and reproductive capacity [22]. In this study, CTX was used to induce POF in 6- to 8-week-old female ICR mice. Successful establishment of the CTX-induced POF mouse model was confirmed by serum hormone measurements and evaluation of ovarian follicle counts. Following UC-MSC transplantation, a significant decrease in FSH levels (P < 0.01). Moreover, the levels of E2, AMH, and P4 in the mouse serum significantly recovered after UC-MSC transplantation (E2: P < 0.01; P4: P < 0.01; AMH: P < 0.01, Additional file 1: Fig. S7B). Histological staining of mouse ovarian tissues with hematoxylin and eosin (H&E) revealed a significant increase in the total number of ovarian follicles in the mice transplanted with human UC-MSCs (P < 0.05, Additional file 1: Fig. S7C, D). These findings indicate that human UC-MSC transplantation can significantly restore ovarian function and reserve in mice with CTX-induced POF. The LRP1^high^ subpopulation increased the number of ovarian follicles in the CTX-induced POF mice (P < 0.05, Fig. 4D, E). However, the LRP1^low^ subpopulation did not exhibit a significant restorative effect, suggesting that compared to the LRP1^low^ subpopulation, the LRP1^high^ subpopulation has a more pronounced restorative effect on the number of ovarian follicles in CTX-induced POF mice. Furthermore, the LRP1^high^ subpopulation remarkably restored in the number of embryos (P < 0.05, Fig. 4F). These results collectively indicate that human UC-MSCs, especially the LRP1^high^ subpopulation, can improve the fertility rate of aged mice or CTX-induced POF mice and can significantly increase the number of high-quality embryos.

### The LRP1^high^ subpopulation of human UC-MSCs can efficiently improve the quality of oocytes

**Fig. 5:**
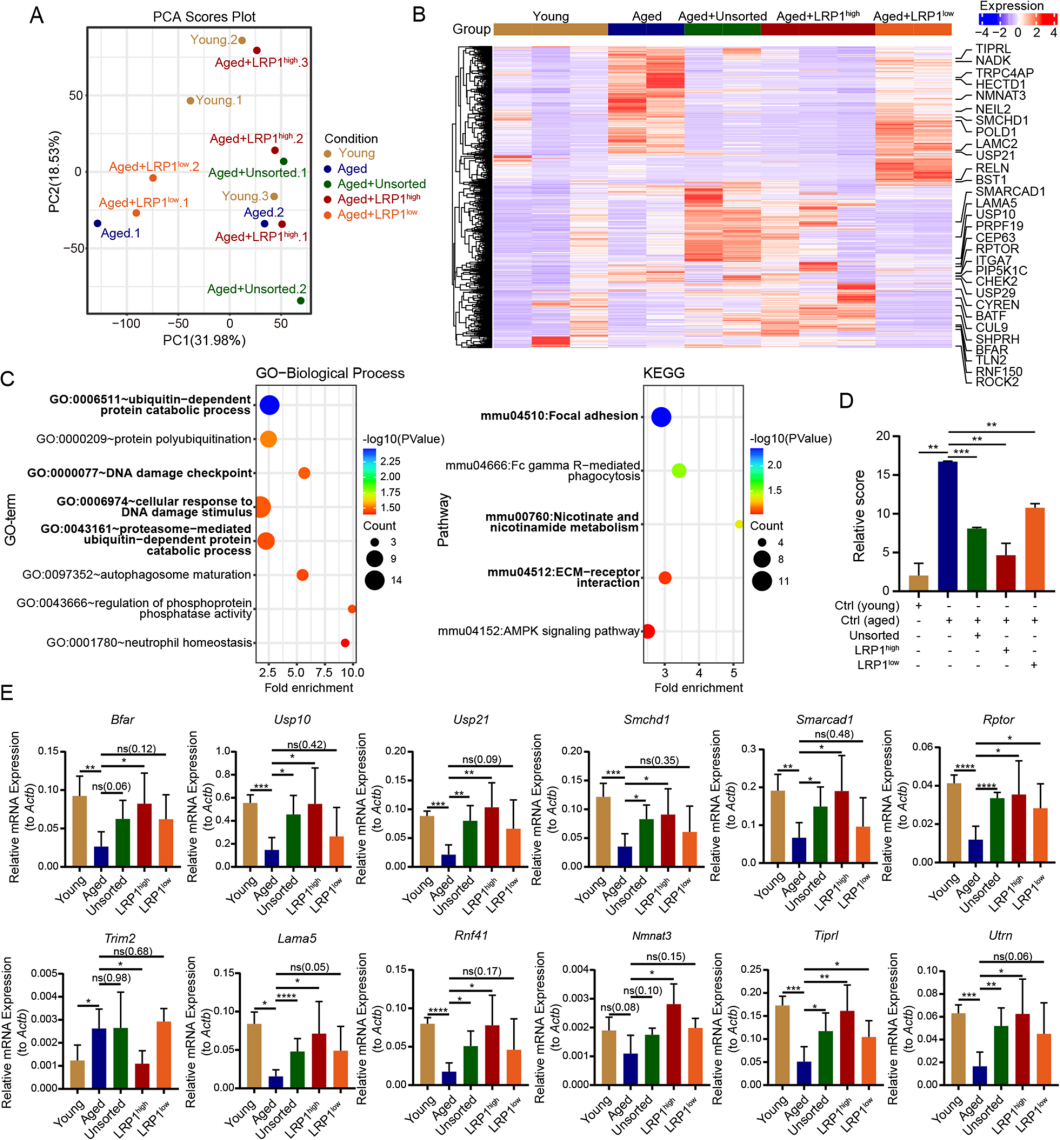
Rescue of oocyte senescence in aged mice by LRP1^high^ subpopulation. **A** PCA analysis demonstrates the transcriptome sequencing results of oocytes from young mice, aged mice, and aged mice with intraovarian transplantation of LRP1^high^ or LRP1^low^ subpopulation. **B** The heatmap displays the differentially expressed genes in oocytes from each group of mice. **C** The GO-BP and KEGG functional enrichment analysis of differentially expressed genes in oocytes from young and aged mice. **D** The oocyte aging scoring system was used to assess the degree of oocyte aging in each group of mice. The transplantation of LRP1^high^ subpopulation in aged mice effectively restored the level of oocyte aging, making it closer to that of young mice. Mean ± SD, *** P ≤ 0.001; ** P ≤ 0.01. **E** At the transcriptional level, the expression of aging genes in oocytes was evaluated after transplantation of different subpopulations of human UC-MSCs. Mean ± SD, n = 3, ** P ≤ 0.01; * P ≤ 0.05. The results showed that LRP1^high^ subpopulation could restore the expression of various pathway-related genes in oocytes of aged mice

To explore the potential mechanisms by which the LRP1^high^ subpopulation improves ovarian function, we performed RNA sequencing analysis on oocytes and granulosa cells from mice transplanted with different subpopulations. The oocyte transcriptome sequencing included a total of five experimental groups: young mouse group (Young), aged mouse group (Aged), aged mice transplanted with unsorted human UC-MSCs (Aged + Unsorted) or LRP1^high^ subpopulation (Aged + LRP1^high^) or LRP1^low^ subpopulation (Aged + LRP1^low^). The results revealed significant differences in gene expression profiles among the groups (Fig. 5A, B), and GO and KEGG analysis revealed that genes exhibiting differential expression in aging oocytes of aged mice compared to young mice were mainly associated with ubiquitin-dependent metabolic pathways, DNA damage checkpoint and response to DNA damage, cell adhesion and extracellular matrix-related signaling, NAM metabolism, ubiquitin-protein ligase activity, and other related pathways (Fig. 5C and Additional file 1: Fig. S8A).

To further investigate the differences in the profiles of oocytes between the various UC-MSC transplantation groups and both young and aged mice, we established a scoring system (Score) using the expression levels of 570 DEGs between young and aged group (Additional file 6: Table S5) to evaluate the oocyte aging status. The oocyte aging status of each subgroup transplantation group was scored to assess the degree of oocyte aging in different groups. Compared to oocytes from young mice, oocytes from aged mice exhibited significant aging (P < 0.01). In the aged mice transplanted with the LRP1^high^ subpopulation, there was a more significant restoration of oocyte status (P < 0.01, Fig. 5D). In the Aged + LRP1^high^ group, a total of 381 aging-related DEGs (66.84%) were restored, which was significantly higher than the restoration observed in Aged + Unsorted group (106 DEGs, 18.60%) and Aged + LRP1^low^ subpopulation group (99 DEGs, 17.37%, Additional file 1: Fig. S8B). This finding indicates that the LRP1^high^ subpopulation had a more pronounced effect on improving the aging status of oocytes in aged mice. Further analysis of the functional pathways regulated or improved by each subgroup transplantation revealed that the 106 genes restored by unsorted UC-MSC were mainly enriched in signaling pathways related to protein binding and protein transport (Additional file 1: Fig. S8C). The 381 genes restored by LRP1^high^ subpopulation were primarily associated with pathways related to NAM metabolism, extracellular matrix and interaction, tight junctions, DNA damage checkpoint and cellular response to DNA damage stimuli, and ubiquitin-dependent protein degradation metabolism (Additional file 1: Fig. S8D). However, the 99 genes restored by LRP1^low^ subpopulation treatment were mainly enriched in pathways related to NAD^+^ kinase activity, oxidoreductase complex, and NADH dehydrogenase complex assembly (Additional file 1: Fig. S8E). These results indicate that the genes restored by LRP1^high^ subpopulation functionally align with the regulatory functions of aging-related genes in aged mice. This finding suggests that LRP1^high^ subpopulation has a highly effective role in restoring the aging status of oocytes in aged mice.

To further validate the impact of the LRP1^high^ subpopulation on gene expression in the oocytes of aged mice, we selected a subset of DEGs with high baseline expression and representative signaling pathways for analysis. The results showed that LRP1^high^ subpopulation restored the expression of multiple genes in the oocytes of aged mice. These included ECM-related genes such as *Lama5* and *Utrn*, DNA damage response-related genes such as *Rptor*, *Smchd1*, *Smarcad1*, *Usp10*, and *Tiprl*, ubiquitin-dependent protein metabolism-related genes such as *Usp21*, *Bfar*, *Rnf41*, and *Trim2*, and the NAD metabolism-related gene *Nmnat3* (Fig. 5E and Additional file 1: Fig. S8F). Importantly, the effects were superior to those observed with unsorted UC-MSCs and the LRP1^low^ subpopulation, indicating that the LRP1^high^ subpopulation may efficiently restore the aging state of oocytes in aged mice through improvements in the ECM, DNA damage associated with aging, and other pathways.

### The LRP1^high^ subpopulation of human UC-MSCs can effectively improve granulosa cell mitochondrial function

In the follicles, granulosa cells surrounding oocytes are the critical somatic component of the ovary. In addition to investigating the improvement of oocyte function by the LRP1 subpopulation of UC-MSCs, we also explored the functional improvement of granulosa cells. Transcriptomic sequencing of granulosa cells included samples from the young mice group, the aged mice group, and the unsorted UC-MSC, or LRP1^high^ subpopulation, or LRP1^low^ subpopulation transplanted group of aged mice. Sequencing analysis revealed that the expression levels of many genes highly expressed in the granulosa cells of aged mice were decreased after LRP1^high^ or LRP1^low^ subpopulation transplantation **(**Additional file 1: Fig. S9A). Previous studies have shown that mitochondrial function in granulosa cells affects ovarian function. To investigate the effects of different subpopulations on mitochondria in senescent granulosa cells induced by CTX, we established a coculture system using human granulosa cell tumor cells (KGN) and UC-MSCs (Additional file 1: Fig. S9B). qRT-PCR results showed the expression level of mitochondrial dynamics genes such as MFF, MFN1, MFN2, TOM20, TIM17A, and TIM17B, and mitochondrial NAD^+^ transporter gene SLC24A17 were significantly upregulated in KGN cells cocultured with the LRP1^high^ subpopulation. In contrast, there were no significant changes in the expression of these genes when cocultured with the unsorted or LRP1^low^ subpopulation (Additional file 1: Fig. S9C). The measurement of mitochondrial membrane potential also indicated that LRP1^high^ subpopulation significantly restored mitochondrial function in CTX-damaged granulosa cells, and the effect was better than that of unsorted and LRP1^low^ subpopulation (Additional file 1: Fig. S9D), indicating UC-MSCs, especially the LRP1^high^ subpopulation could restore ovarian function through regulating mitochondrial function in granulosa cells.

## Discussion

To date, human UC-MSCs possess characteristics such as convenient accessibility and noninvasive collection, making them an ideal seed cell for the clinical treatment of POF [9, 23]. However, the considerable heterogeneity observed in human UC-MSCs isolated or long-term cultured in vitro has greatly hindered their large-scale research and clinical application [24–26]. Therefore, in-depth exploration of the heterogeneity of human UC-MSCs and study of the therapeutic effects of different functional subpopulations on POF have major clinical implications in identifying specific subpopulation that efficiently improve ovarian function. Here, based on the single-cell heterogeneity analysis on two cases of human UC-MSCs using 10 × Genomics technology, we classified the 16 cell clusters which could distinguish into three cell subtypes, namely, MSCs, MPCs, and trophoblasts. On the basis of DEGs among clusters, we identified LRP1 as a marker to isolate the subpopulation that exhibited high expression and secretion of multiple cytokines, growth factors, and chemokines. Importantly, through ovarian subcapsular transplantation, we confirmed that the LRP1^high^ subpopulation not only restored abnormal hormone levels and shortened estrous cycles in aged mice but also markedly increased the number of follicles in CTX-induced POF mice and improved the quantity and morphology of E12.5 embryos, outperforming the unsorted UC-MSCs and LRP1^low^ subpopulation. Through a comparison of the transcriptome of oocytes and granulosa cells before and after transplantation, we found that LRP1^high^ subpopulation remarkably regulated pathways related to ECM, DNA damage and NAM metabolism in the oocytes of aged mice. Furthermore, through co-culture system, we found that the LRP1^high^ subpopulation can activate the mitochondrial dynamics genes and there is also a significant increase in the expression of the mitochondrial NAD transporter gene SLC24A17, indicating that the LRP1^high^ subpopulation may also restore ovarian function by improving NAD metabolism and restoring mitochondrial dynamics within granulosa cells.

The high heterogeneity of stem cells is a major factor contributing to unfavorable clinical outcomes [17]. Not only is there heterogeneity among stem cells derived from different tissues, but variability across different generations and samples also significantly affects the reproducibility of stem cell therapies [15, 16]. In this study, we employed single-cell technology to analyze the heterogeneity of two P6 generation UC-MSCs derived from the same tissue source, identifying three distinct cell types. The results indicated the presence of two subtypes, MPCs and trophoblasts, which had not been explicitly identified in previous studies, in addition to MSCs. Similarly, Zhang et al. utilized single-cell RNA sequencing on MSCs from bone marrow and Wharton's jelly, discovering a subgroup identified as chondrocyte precursor cells that exhibited specific expression of immune regulatory genes. This subgroup demonstrated the ability to inhibit the proliferation of activated CD3^+^ T cells in vitro, supporting its potential role in immune regulation [27]. Additionally, other studies have suggested the existence of a subgroup within UC-MSCs that expresses genes related to immune response and regulation. These findings highlight the remarkable immunomodulatory capabilities of UC-MSCs [18]. Previous studies have indicated that UC-MSCs from different gender sources may also exhibit cellular-level heterogeneity. Gender differences play a decisive role in certain functions of MSCs, particularly in immunomodulation [28, 29]. Similarly, in our data, we have observed a similar gender-dependent gene expression phenomenon (data not shown). A more crucial observation is that, consistent with the results obtained from external validation data, there is significant heterogeneity among UC-MSCs from different sample sources. This heterogeneity may be attributed to the fact that the samples are derived from single cells rather than tissues [30]. Due to the substantial heterogeneity observed in human UC-MSCs, we are motivated to further investigate their heterogeneity and urgently search for highly specific subpopulations of human UC-MSCs that play a crucial therapeutic role in POF.

LRP1, low-density lipoprotein receptor-related protein 1, is a member of the low-density lipoprotein receptor family and can bind to over 100 ligands, including matrix metalloproteinases, urokinase-type plasminogen activator, transforming growth factor-β1, and apolipoprotein E, playing crucial roles in controlling inflammation, tissue remodeling, and extracellular molecule clearance [31–36]. Disruption of the LRP1 gene in mice prevents the development of LRP1^−/−^ embryos during the implantation stage, highlighting the importance of LRP1 in developmental physiology [37]. In our study, we identified a subpopulation marked by LRP1 that is unrelated to the heterogeneity of the three aforementioned cell subtypes. Consistent with prior research, the LRP1^high^ subpopulation is significantly associated with ECM functionality and the secretion of regulatory factors [38–42]. The ECM within the ovaries is complex, and age-related changes in ECM homeostasis, characterized by increased collagen synthesis and decreased hyaluronic acid deposition, significantly impact follicle growth and the quality of oocytes [43–46]. Additionally, the LRP1-mediated serine protease and MMP clearance mechanism regulates extracellular protein degradation activity and controls extracellular MMP levels, thereby altering cell adhesion and migration abilities [47, 48]. In vivo, we observed that the LRP1^high^ subpopulation significantly downregulated ECM-related pathway genes, such as MMP12, COL26A1, and LAMC2, in the oocytes of aged mice. This result indicates that the LRP1^high^ subpopulation may contribute to ECM remodeling in aging ovaries or improve oocyte growth and function.

Previous studies have shown that MSCs can localize to the ovarian stroma and secrete various cytokines, thereby improving ovarian reserve function through paracrine pathways, which is a key factor in restoring ovarian function [49, 50]. Various cytokines secreted by the LRP1^high^ subpopulation, particularly FGF7, have been shown to promote ECM synthesis and facilitate endogenous repair [51]. C–C chemokines such as CCL2 can stimulate integrin-mediated ECM assembly to protect oocytes [52]. In this study, we found that the LRP1^high^ subpopulation, while expressing TGFBI, is involved in the regulation of the TGF-β signaling pathway. TGF-β belongs to the cysteine knot superfamily, including the glycoprotein hormone family (such as follicle-stimulating hormone, luteinizing hormone, and thyroid-stimulating hormone), and the platelet-derived growth factor family, among others [53]. It promotes the growth and differentiation of target tissues from early embryonic development and is widely involved in paracrine effects in adult tissues [54]. Previous studies have demonstrated that hAMSCs significantly inhibit chemotherapy-induced cell apoptosis and activate the TGF-β/Smad signaling pathway within primary granulosa cells in a paracrine manner [55]. This suggests that the TGF-β signaling associated with the LRP1^high^ subpopulation plays a crucial role in significantly improving ovarian function. Given the robust paracrine effects of LRP1^high^ UC-MSCs, in the future, we will further delineate and screen for the primary secretion factors of LRP1^high^ subpopulation. We aim to utilize these potential factors individually or in combination for ovarian subcapsular or intraperitoneal injections, selecting specific cytokines, growth factors, and combinations that can repair ovarian function without the need for UC-MSCs transplantation, thus avoiding potential side effects. This approach may also lay the foundation for the future large-scale production of remedies to restore ovarian function or cocktail therapies.

Oxidative stress is closely associated with tissue damage and aging, and it is one of the causes of POF [56]. Mitochondria are the primary site of reactive oxygen species (ROS) production and are susceptible to ROS-induced damage [57]. Additionally, mitochondria serve as the powerhouses of various cells, including human oocytes and granulosa cells. Elevated levels of ROS in oocytes lead to telomere shortening and decreased developmental capacity, while high ROS levels disrupt communication between oocytes and granulosa cells, affecting oocyte maturation before ovulation [58, 59]. MSCs can exert antioxidant effects by secreting various cytokines and extracellular vesicles, promoting the generation of antioxidant enzymes and inhibiting the production of ROS, thereby alleviating oxidative stress and improving ovarian function [60, 61]. We found that the LRP1^high^ subpopulation can regulate pathways related to DNA damage and NAM metabolism within oocytes of aged mice. In particular, the DNA damage response checkpoint pathway, represented by genes such as CHEK2, TIPRL, CEP63, and PRPF19, and the NAM metabolic pathway, represented by BST1, NMNAT3, and NADK, showed significant improvements. Furthermore, the LRP1^high^ subpopulation can activate the expression of the mitochondrial dynamics-related genes MFF, MFN1, MFN2, TOM20, TIM17A, and TIM17B in KGN cells in vitro, and there is also a significant increase in the expression of the mitochondrial NAD transporter gene SLC24A17. These findings suggest that the LRP1^high^ subpopulation may restore ovarian function by improving NAD metabolism within oocytes and restoring mitochondrial dynamics within granulosa cells. Further research is needed to elucidate the specific mechanisms involved.

## Conclusion

In summary, this study revealed the heterogeneity of human UC-MSCs using 10 × Genomics single-cell sequencing technology. Through in vivo and in vitro experiments, it was demonstrated that the LRP1^high^ subpopulation has therapeutic effects in improving ovarian function. Furthermore, mechanistic exploration revealed that the LRP1^high^ subpopulation primarily improves the quality of aged mouse oocytes through DNA damage pathways, extracellular matrix-related signaling, and regulation of cellular metabolism. Additionally, it enhances the mitochondrial function of granulosa cells. Thus, this specific LRP1^high^ subpopulation we identified holds promise for precise stem cell therapy in POF, improving ovarian function in POF patients and providing new insights into mitigating the instability associated with stem cell heterogeneity.

### Supplementary Information

**Additional file 1.** Supplementary Figures S1–S9.

**Additional file 2. Table S1.** Marker of cell subtypes (Related to Fig. 1).

**Additional file 3. Table S2.** Information of subtype and cluster of UC-MSCs. (Related to Fig. 1).

**Additional file 4. Table S3.** DEGs of each sub-cluster. (Related to Fig. 1).

**Additional file 5. Table S4.** DEGs of LRP1high vs LRP1low subpopulation (Related to Fig. 2).

**Additional file 6. Table S5.** Aging score of each sample (Related to Fig. 5).

**Additional file 7. Table S6.** Primer's sequence of genes used in this study (Related to Fig. 2, 3, 5, Additional file 1: Fig. S3, S9).

## Data Availability

The sequencing data sets have been deposited in NCBI’s Gene Expression Omnibus (GEO) and are accessible through the GEO accession number GSE239500 and GSE240185. The external validation dataset GSE199071 was download from GEO.

## Abbreviations

AMSCs: Amniotic mesenchymal stem cells
ADSCs: Adipose-derived stem cells
BM-MSCs: Bone marrow-derived mesenchymal stem cells
BP: Biological process
CC: Cellular component
CTX: Cyclophosphamide
DEGs: Differentially expressed genes
ECM: Extracellular matrix
GO: Gene ontology
HRT: Hormone replacement therapy
KEGG: Kyoto encyclopedia of genes and genomes
LRP1: Low-density lipoprotein receptor-related protein 1
MF: Molecular function
MPCs: Multilymphoid progenitor cells
MSCs: Mesenchymal stem cells
PCA: Principal component analysis
PMSCs: Placental mesenchymal stem cells
POF: Premature ovarian failure
ROS: Reactive oxygen species
UC-MSCs: Umbilical cord-derived mesenchymal stem cells
