# Supplementary material for: Transplantation of the LRP1high subpopulation of human umbilical cord-derived mesenchymal stem cells improves ovarian function in mice with premature ovarian failure and aged mice
Source: Stem Cell Res Ther. 2024 Mar 5;15:64. doi: 10.1186/s13287-024-03660-0 (PMC10913679; doi:10.1186/s13287-024-03660-0)
Supplement: Supplementary file 1 — Additional file 1. Supplementary Figures S1–S9. [file 13287_2024_3660_MOESM1_ESM.pdf]

Supplementary figure 1

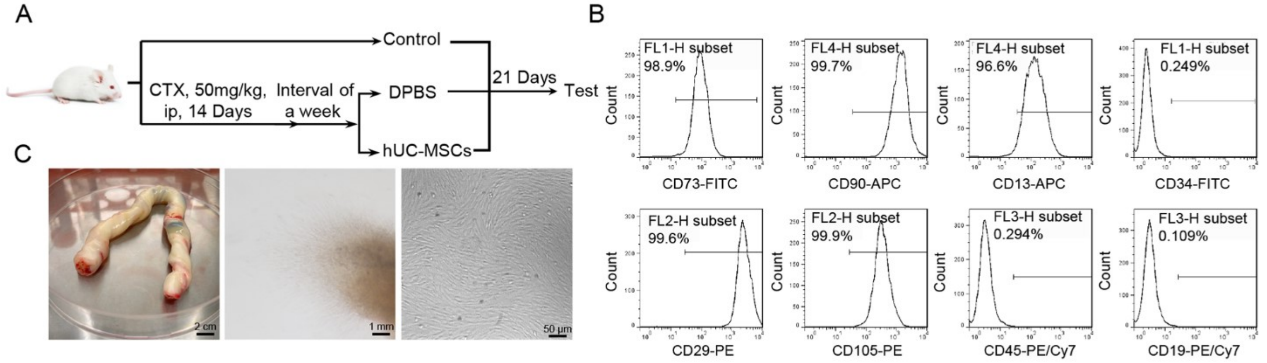

Fig. S1 Isolation, identification of human UC-MSCs, CTX-induced POF mouse modeling protocol (Related to Fig. 1 and 4). A Method for establishing the CTX-induced POF mouse model and evaluation of ovarian function after human UC-MSCs transplantation. **B** Isolation of human UC-MSCs. Human UC-MSCs were isolated from umbilical cord tissues obtained from different fetuses (top panel). In the P0 generation, human UC-MSCs can be observed migrating out from the edges of tissue fragments (middle panel, 5x magnification). The P1 passage of human UC-MSCs is shown in the panel below (20x magnification). **C** The primary human UC-MSCs were identified using stem cell markers CD73 (98.9%), CD90 (99.7%), CD13 (96.6%), CD29 (99.6%), and CD105 (99.9%), and hematopoietic cell markers CD19 (0.249%), CD34 (0.294%), and CD45 (0.109%).

Supplementary figure 2

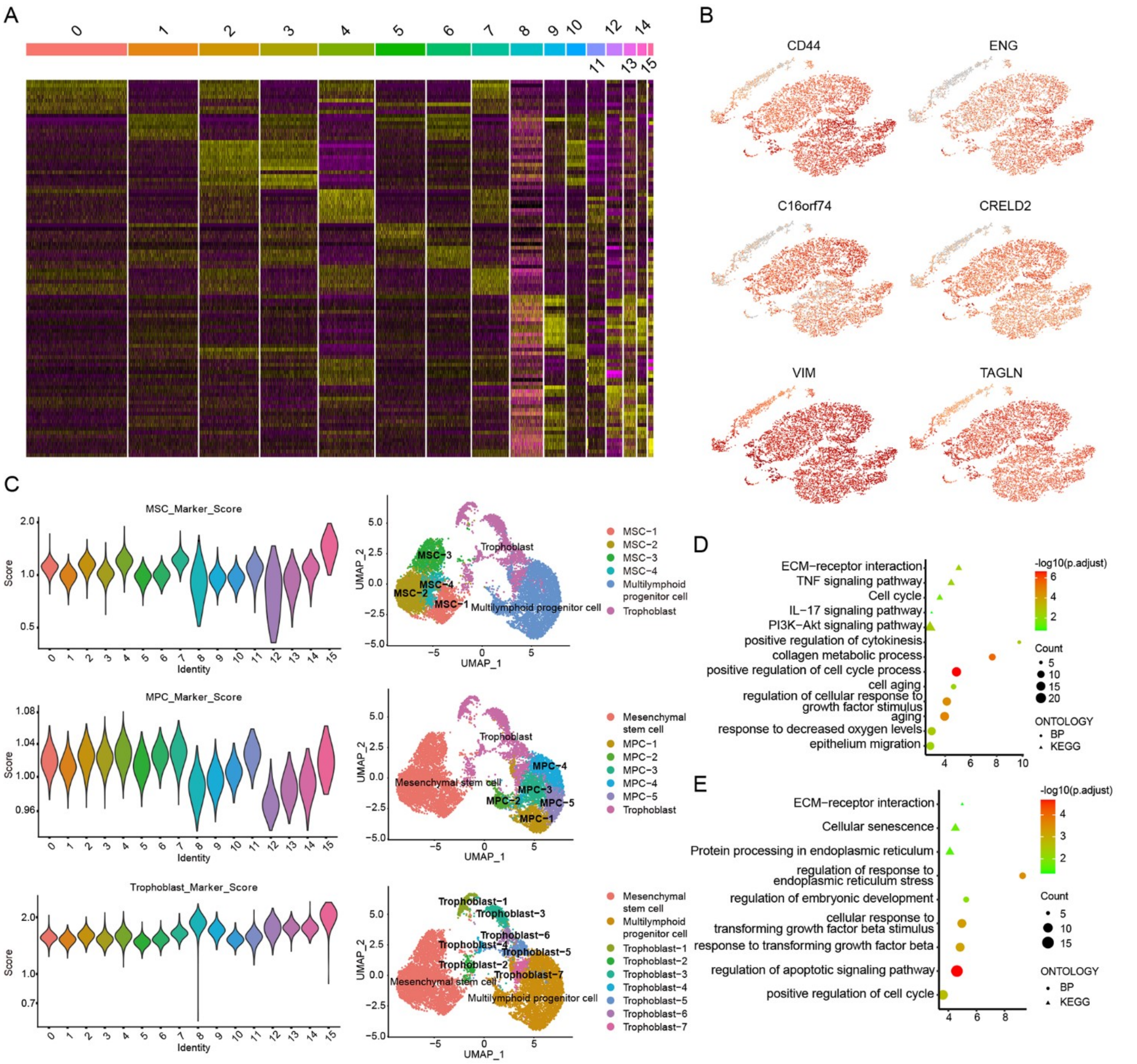

Fig. S2 The heterogeneity of human UC-MSCs at single-cell resolution and function enrichment of subtypes (Related to Fig. 1). A Displaying the heatmap of differentially expressed genes among clusters of cells. **B** The cLoupe software displays the expression levels and distribution of representative marker genes for three subtypes of human UC-MSCs. **C** The expression levels of marker genes for each subpopulation in each cluster of cells and the subgroup subdivision within each subtype. **D** The functional pathways (represented by GO-BP and KEGG) regulated by MSCs subtype. **E** The functional pathways (represented by GO-BP and KEGG) regulated by MPCs subtype.

Supplementary figure 3

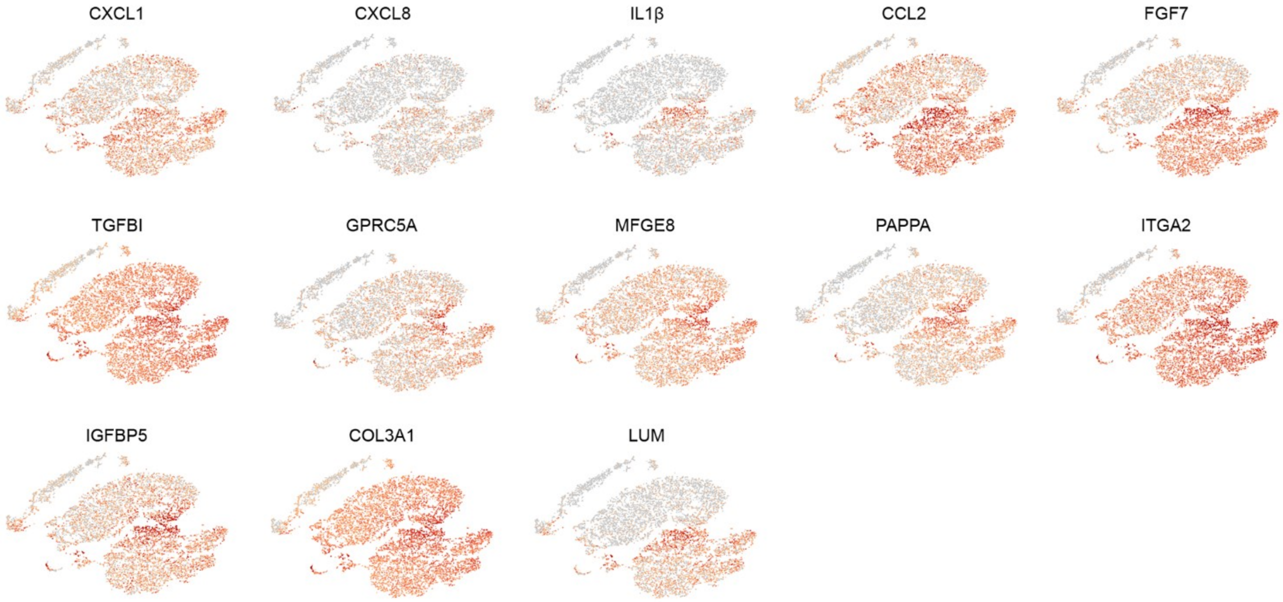

Fig. S3 LRP1^{high} is the subpopulation with high secretory function (Related to Fig. 3). cLoupe displays the expression levels and distribution of LRP1, related secretory factors, and ECM-related genes.

Supplementary figure 4

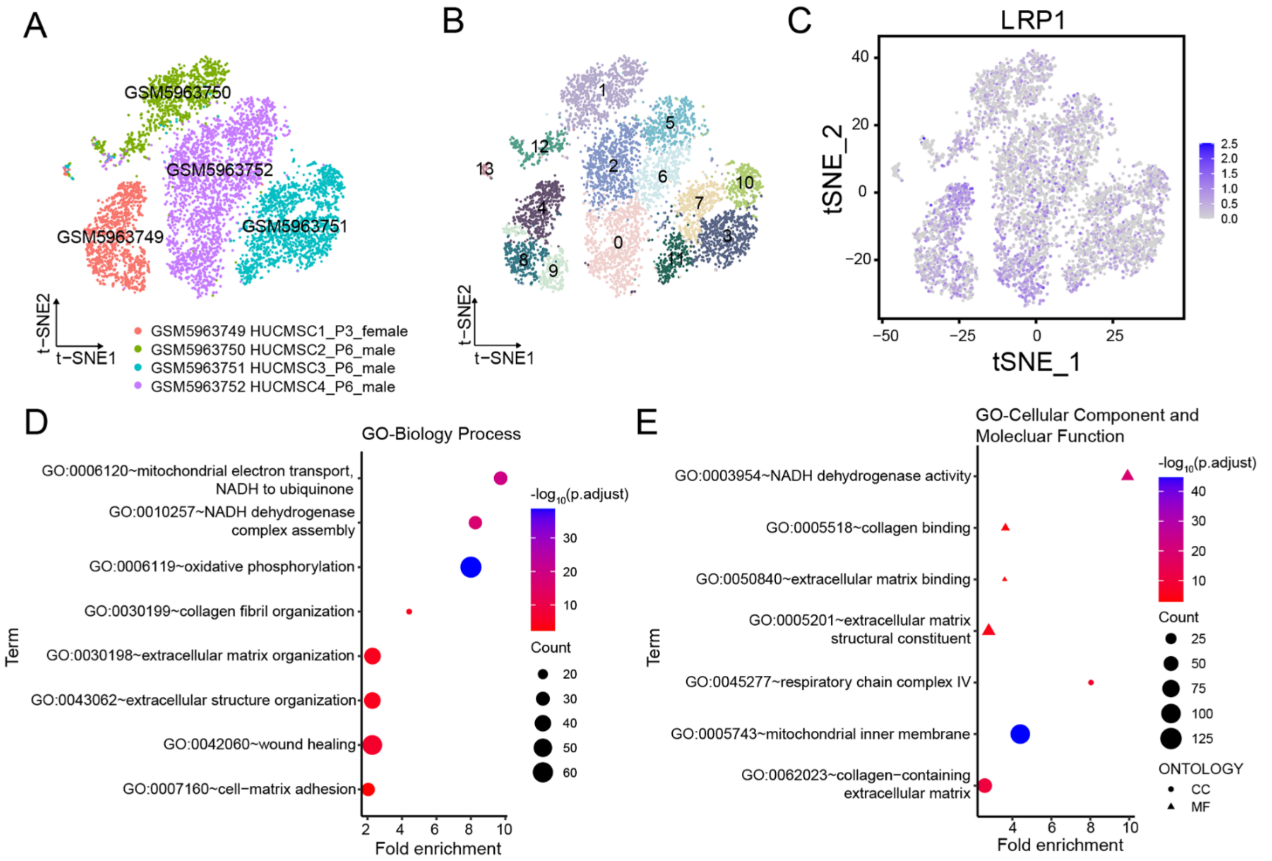

Fig. S4 External validation of the LRP1 function analysis (Related to Fig. 2). A t-SNE plot displays the clustering information of four cases of human UC-MSCs, distinguished by sample. **B** t-SNE plot visualization of cluster information of 14 subclusters of four cases of human UC-MSCs. **C** t-SNE plot visualization of the expression levels of LRP1. **D** Functional enrichment analysis (GO-BP) of LRP1 related regulatory pathways. **E** Functional enrichment analysis (GO-CC and GO-MF) of LRP1 related regulatory pathways.

Supplementary figure 5

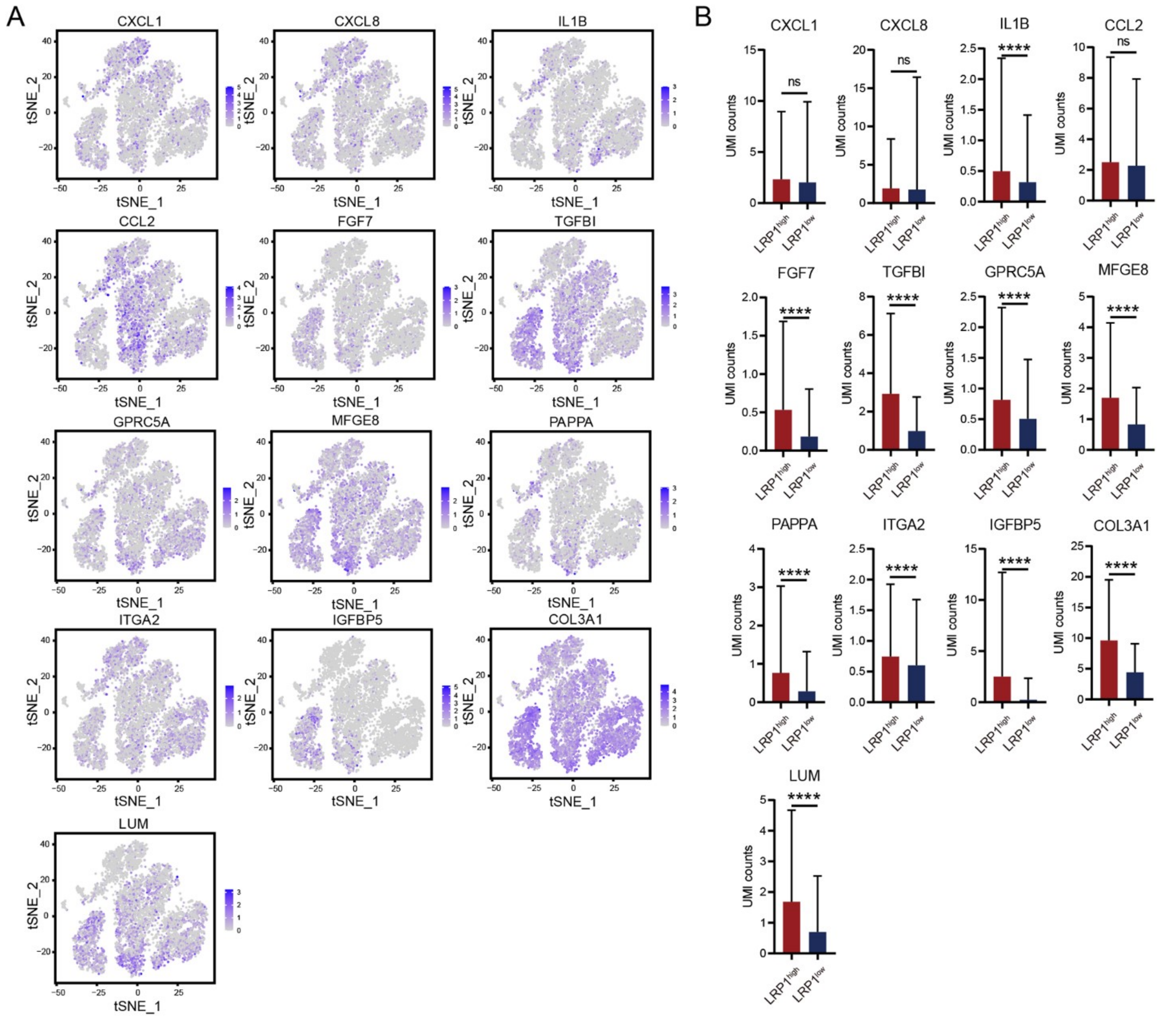

Fig. S5 External validation of functional characterization of the LRP1^{high} subpopulation (Related to Fig. 3). A t-SNE plot visualization of the expression levels of secreted factors and extracellular matrix related genes. **B** Bar plot visualization of expression levels of secreted factors and extracellular matrix related genes grouped by LRP1 expression.

Supplementary figure 6

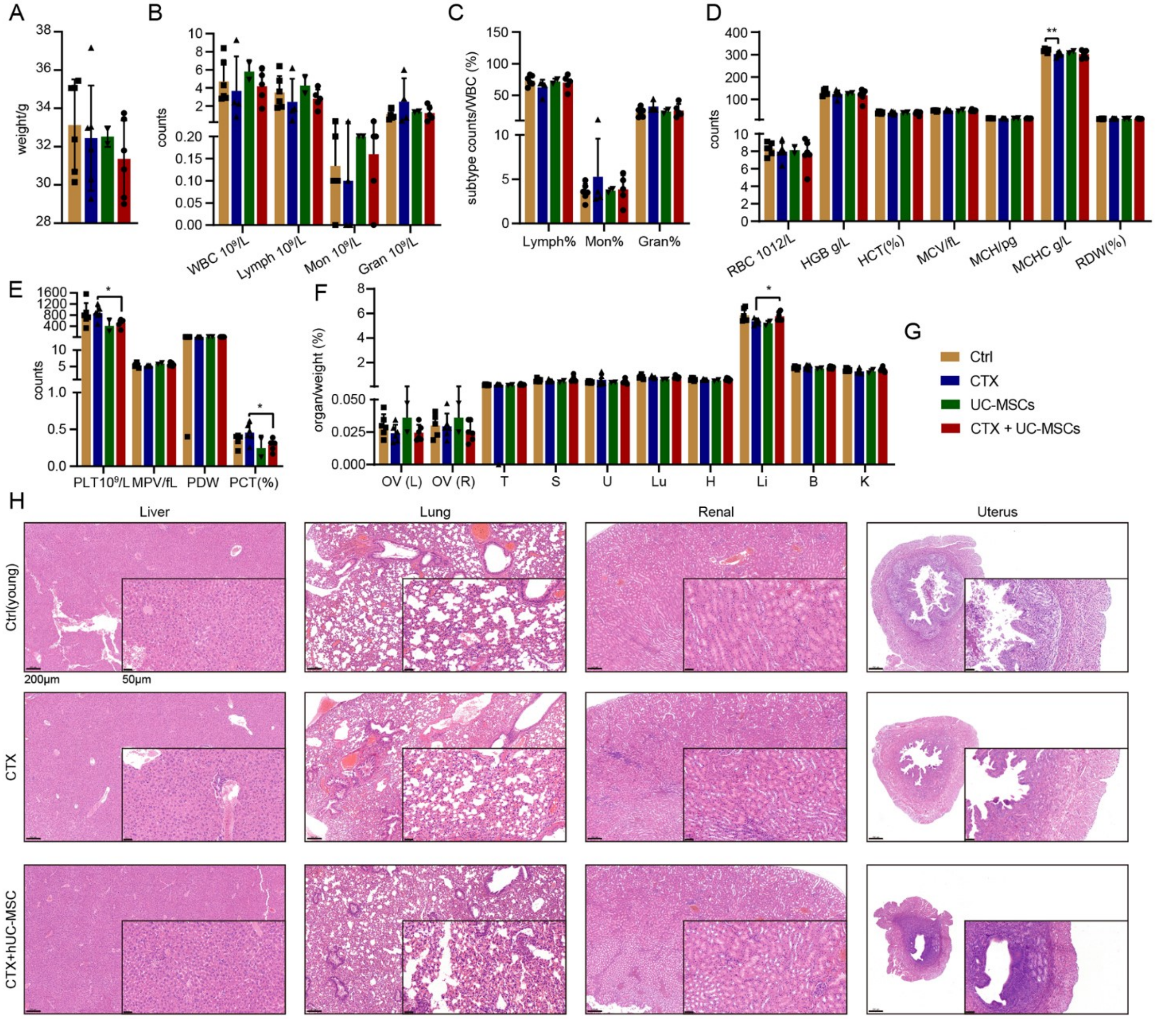

Fig. S6 Evaluation of safety, effectiveness of the human UC-MSCs transplantation (Related to Fig. 4). A Weight of mice in Ctrl (control), CTX treatment, human UC-MSCs transplantation and CTX+UC-MSCs group. There was no significant difference between the groups. The corresponding color of each group is shown in **G**. **B-E** The indexes of peripheral blood cells of mice in each group were detected. The detection indicators were WBC, Lymph, Mon, Gran (**B**), Lymph%, Mon%, Gran% (**C**), RBC, HGB, HCT, MCV, MCHC, RDW (**D**), PLT, MPV, PDW, PCT (**E**). Mean±SD, $**p \leq 0.01$; $*p \leq 0.05$. The corresponding color of each group is shown in **G**. **F** Body weight ratio of organs in each group of mice. Mean±SD, $*p \leq 0.05$. The corresponding color of each group is shown in **G**. **G** Corresponding to the groups in panel **A-F**. **H** Assessment of the safety of human UC-MSCs intraovarian transplantation therapy through H&E staining of various mouse organ tissues.

Abbreviations: WBC, white blood cell; Lymph, lymphocyte; Mon, monocyte; Gran, granule cell; RBC, red blood cell; HGB, haemoglobin; HCT, hematocrit; MCV, mean corpuscular volume; MCHC, mean corpuscular hemoglobin concentration; RDW, red blood cell volume distribution width; PLT, platelet; MPV, Mean platelet volume; PDW, platelet volume distribution width; PCT, plateletocrit; OV (L), ovarian (left); OV (R), ovarian (right); T, thymus; S, spleen; U, uterus; Lu, lung; H, heart; Li, liver; B, brain; K, kidney.

Supplementary figure 7

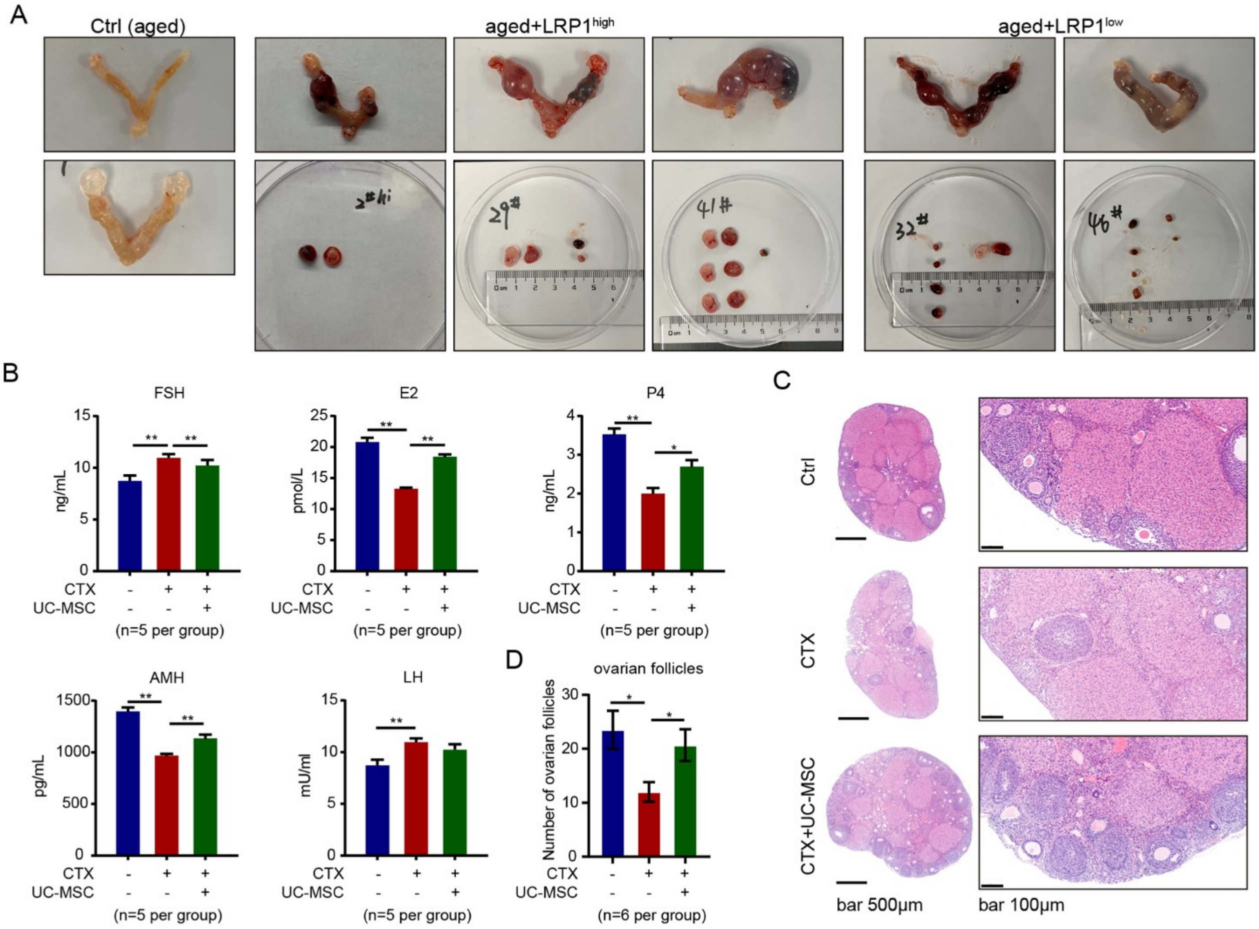

Fig. S7 LRP1^{high} subpopulation significantly restored ovarian function in aged mice and evaluation of the results of CTX-induced POF mouse modeling (Related to Fig. 4). A Detection of E12.5 embryonic pregnancy status in aged mice receiving transplantation of LRP1^{high} and LRP1^{low} subpopulations. **B** The levels of FSH, LH, E2, P4 and AMH in the peripheral blood serum of CTX-induced POF mice and POF mice treated with human UC-MSCs. Mean±SD, **$p \leq 0.01$; *$p \leq 0.05$. **C, D** Quantification of the number of follicles at different stages in the ovaries of CTX-induced POF mice and POF mice treated with human UC-MSCs, mean±SD, *$p \leq 0.05$.

Supplementary figure 8

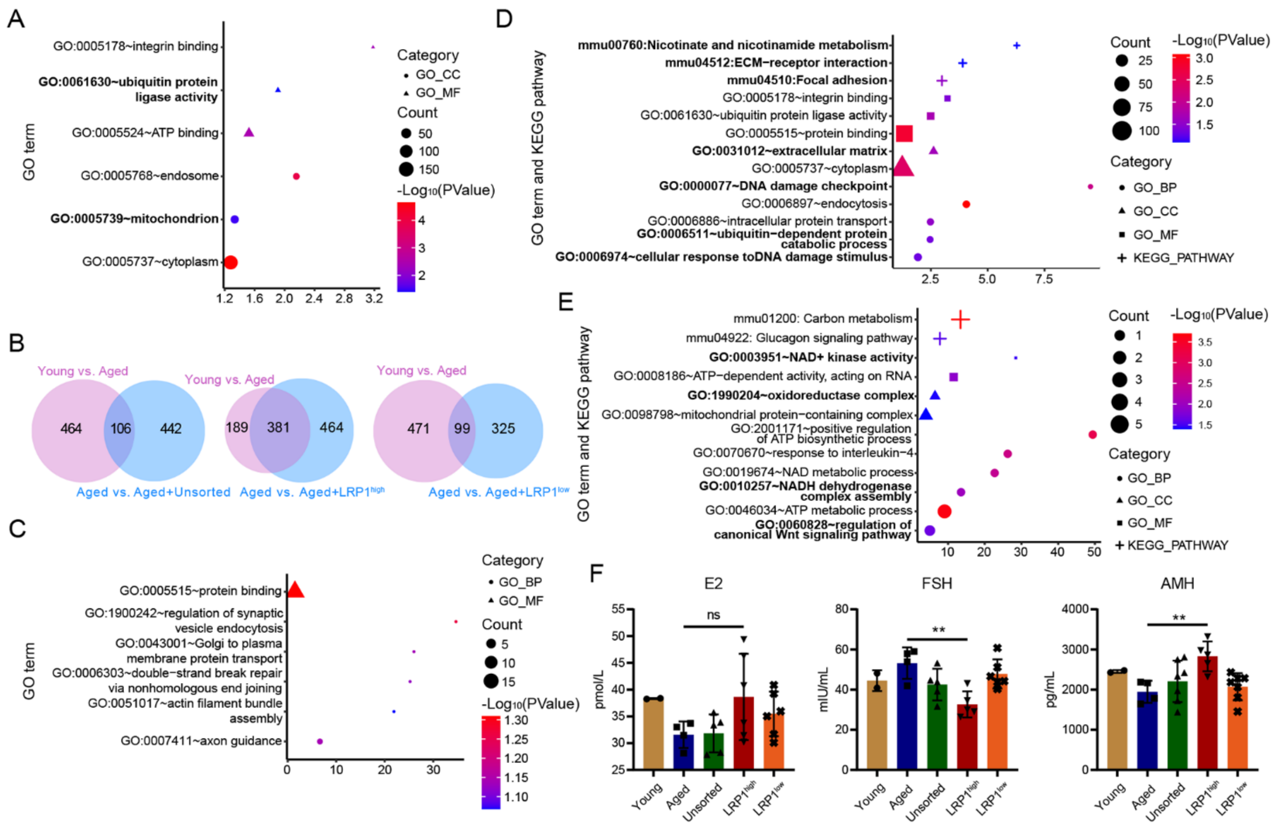

Fig. S8 The regulation of oocyte and granulosa cell expression profiles in aged mice by different subpopulations of human UC-MSCs (Related to Fig. 5). A The GO-CC and GO-MF functional enrichment analysis of DEGs in oocytes from young and aged mice. **B** The impact of each subpopulation of human UC-MSCs on the number of aging genes in oocytes of aged mice. **C** The functional enrichment of restored aging genes in oocytes after treatment with unsorted human UC-MSCs. **D** The functional enrichment of restored aging genes in oocytes after treatment with LRP1^{high} subpopulation. **E** The functional enrichment of restored aging genes in oocytes after treatment with LRP1^{low} subpopulation. **F** The serum hormone levels of the validation batch of mice were tested to ensure the effectiveness of the modeling and human UC-MSCs subpopulation transplantation. The number of mice used was represented by different shaped dots in each group. Mean±SD, $**p \leq 0.01$; ns: $p > 0.05$.

Supplementary figure 9

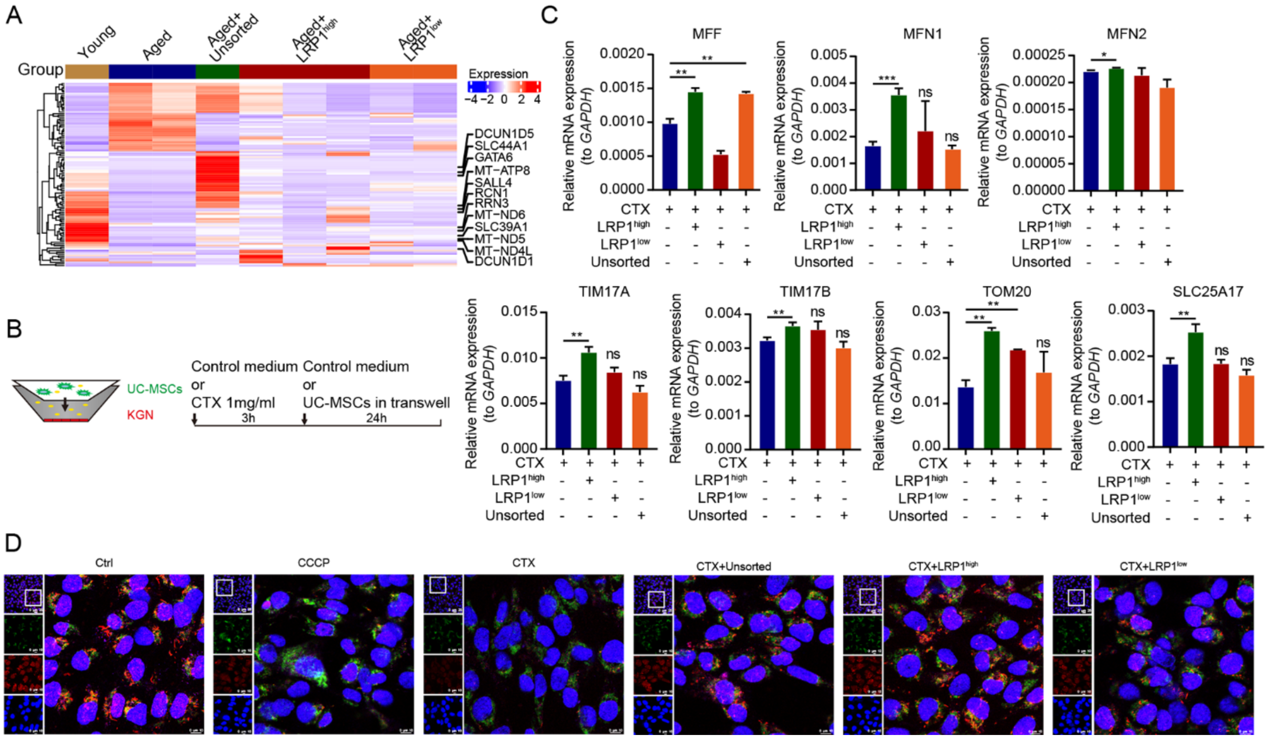

Fig. S9 The LRP1^{high} subpopulation more efficiently improves the mitochondrial function of ovarian granulosa cells. A The heatmap displays the DEGs in granulosa cell from each group of mice. **B** The CTX induction protocol and co-culture model of human granulosa tumor cells (KGN) with human UC-MSCs. **C** The impact of different subpopulations on mitochondrial dynamics genes within granulosa cells, with LRP1^{high} subpopulation significantly restoring the expression of mitochondrial dynamics genes. Mean±SD, ***$p \leq 0.001$; **$p \leq 0.01$; *$p \leq 0.05$; ns: $p > 0.05$. **D** LRP1^{high} subpopulation improve the mitochondrial membrane potential in granulosa cells.
